# Insights into the flavor evolution of braised chicken during six-month storage via sensory evaluation, E-tongue, E-nose, and GC–IMS

**DOI:** 10.1016/j.fochx.2026.104631

**Published:** 2026-10-15

**Authors:** Liu Jing, Li Huanjia, Yue Caishuan, Su Zhiwei, Li Yujin, Wang Bingui, Guo Yujie

**Affiliations:** a Institute of Oceanology, Chinese Academy of Sciences, Qingdao 266071, China; b College of Life Sciences, Dezhou University, Dezhou 253023, China; c Dezhou Xiangsheng Food Co., Ltd., Dezhou 253000, China; d Institute of Food Science and Technology, Chinese Academy of Agricultural Sciences, Beijing 100193, China

**Keywords:** Braised chicken, Flavor evolution, Electronic nose, Electronic tongue, GC-IMS, Shelf-life, 3-Methyl butanal (PubChem CID: 11552), 1-Octanal (PubChem CID: 454), 2,3-Butanedione (PubChem CID: 650), Ethyl 3-methylbutanoate (PubChem CID: 7945), 1-Butanoic acid (PubChem CID: 264), 1,8-Cineole (PubChem CID: 2758), Ethyl methyl disulfide (PubChem CID: 123388), Diethyl disulfide (PubChem CID: 8077), 2-Furanmethanethiol (PubChem CID: 7363)

## Abstract

The flavor of braised chicken tends to deteriorate during prolonged storage. This study systematically investigated dynamic changes in the flavor of braised chicken during six-month storage. Sensory and physicochemical analyses revealed a relatively stable appearance and color, but a noticeable deterioration in taste and texture. Odor degraded most significantly. The electronic tongue (E-tongue) detected a taste shift toward decreased umami and richness and increased saltiness. The electronic nose (E-nose) supported the dominant role of odor by revealing a substantial build-up of sulfur- and nitrogen-containing volatiles. Gas chromatography–ion mobility spectrometry analysis identified 67 volatile compounds in braised chicken during storage. Orthogonal partial least squares–discriminant analysis screening identified ten key differential compounds. Among these, octanal, diethyl disulfide, butanoic acid, and 2-furanmethanethiol showed strong correlations with sensory deterioration and served as potential markers of flavor degradation. This study provides a reference for evaluating the edibility and salability of braised chicken during its shelf-life.

## Highlights

• Flavor evolution of 6-month stored braised chicken was systematically evaluated.
• Odor deterioration was the most pronounced effect among all sensory attributes.
• The taste profile moved toward reduced umami and richness and enhanced saltiness.
• The odor profile was enriched with sulfur- and nitrogen-containing compounds.
• Octanal, diethyl disulfide, butanoic acid, 2-furanmethanethiol as potential markers.

## Graphical abstract

**Figure:**
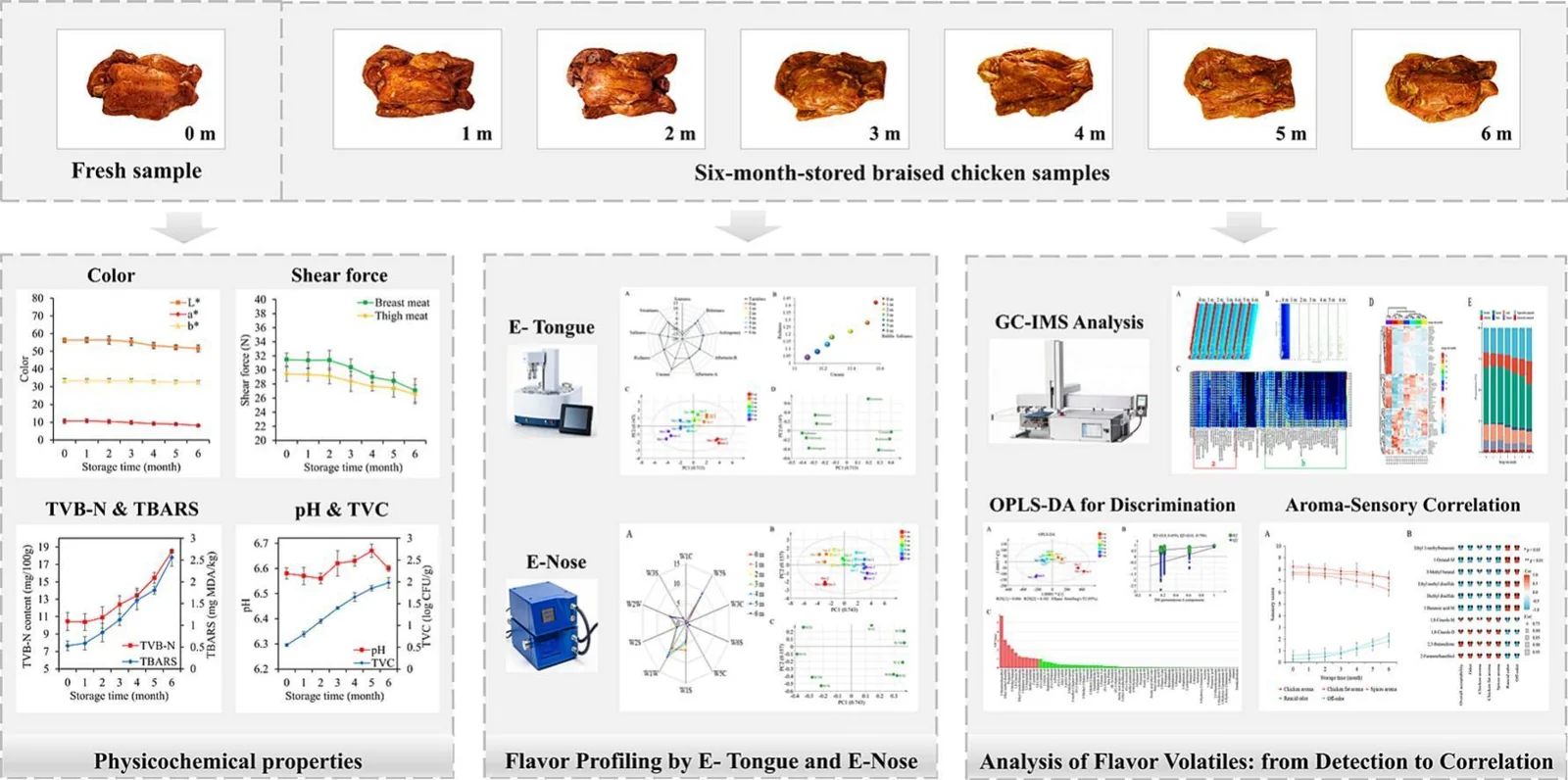

## 1 Introduction

Braised chicken is a traditional and popular ready-to-eat meat product in China. It is renowned for its unique flavor profile, tender texture, and convenience (Duan et al., 2015; Lei et al., 2022). Its characteristic flavor, which is formed through complex interactions among seasonings, spices, and thermal reactions during stewing, is widely recognized as the most critical factor influencing consumer acceptability and market competitiveness (Sun et al., 2024). However, during prolonged storage, the flavor of braised chicken is susceptible to deterioration due to a series of physiochemical and microbiological changes, such as lipid oxidation, protein degradation, and microbial metabolism (Liu et al., 2019; Wang et al., 2025). Specifically, lipid oxidation generates volatile aldehydes and ketones responsible for rancid off-odors, while protein degradation and microbial metabolism produce amines and sulfur-containing compounds that contribute to off-flavors (Bekhit et al., 2021; Tatiyaborworntham et al., 2022). These processes inevitably lead to the loss of desirable flavor compounds, development of off-odors and off-tastes, and, ultimately, a decline in sensory appeal, which significantly shortens the product's shelf-life and reduces consumer satisfaction (Shi et al., 2025; Zhang et al., 2025). Therefore, a comprehensive understanding of the dynamic evolution of flavor compounds during storage is essential not only for establishing effective quality control protocols but also for guiding shelf-life prediction and developing preservation strategies for the braised chicken industry.

Traditionally, flavor evaluation in food systems has primarily relied on trained sensory panelists. Although this approach provides valuable information directly related to human perception, its practical application is often constrained by subjectivity, time consumption, and variability among panelists (Jiao et al., 2022). To overcome these limitations, instrumental analytical methods are being increasingly employed for research on food flavor in recent years. Electronic nose (E-nose) and electronic tongue (E-tongue) systems, which mimic human olfactory and gustatory responses via sensor arrays, enable a rapid and objective preliminary assessment of the overall odor and taste profiles of foods (Huang et al., 2023; Liu et al., 2024). They are particularly suitable for the rapid screening and classification of large sample sets. Gas chromatography–ion mobility spectrometry (GC–IMS) has emerged as a promising analytical technique for volatile organic compounds. GC–IMS, which combines the high separation capability of gas chromatography with the high sensitivity and rapid response of ion mobility spectrometry, allows an efficient separation and identification of flavor compounds in complex food matrices with minimal sample pretreatment (Pan et al., 2025; Wang et al., 2024). This technique is particularly adept at constructing visual flavor fingerprints, providing a powerful tool for tracking dynamic changes in volatile compounds during food processing or storage (Shi et al., 2025; Yao et al., 2025).

The integration of these complementary analytical techniques—E-nose for overall odor profiling, E-tongue for basic taste characterization, and GC–IMS for a precise identification of volatile compounds—collectively establishes a multidimensional analytical framework capable of effectively linking macro-scale sensory attributes with micro-scale chemical compositions. This multitechnique strategy can be used for a comprehensive elucidation of flavor evolution in complex food systems. For instance, an integrated application of HS–GC–IMS, E-nose, and E-tongue has been successfully employed to clarify the volatile flavor and quality profiles of loquat during its shelf-life (Huang et al., 2023), and to evaluate the impact of various processing steps on volatile flavor compounds and their precursors in beer fish production (Liu et al., 2024). However, the systematic application of such an integrated approach to track the flavor trajectory during the long shelf-life of complex-flavored seasoned meat products such as braised chicken remains insufficiently explored. In particular, there is a scarcity of systematic investigations that employ multidimensional data fusion—encompassing sensory, physicochemical, E-nose /E-tongue, and GC–IMS volatile profiles—coupled with chemometric analysis to identify key markers of flavor deterioration.

To address this gap, the present study aims to systematically investigate the dynamic flavor evolution of Dezhou braised chicken over six months of storage using an integrated strategy comprising sensory evaluation, physicochemical analysis, E-tongue, E-nose, and GC–IMS. This stepwise framework—from sensory attributes to E-nose /E-tongue profiles, then to GC–IMS volatiles, and finally to correlation analysis—allows us to pinpoint volatile markers most closely linked to sensory deterioration. Vacuum-packaged braised chicken serves as a representative model for studying flavor deterioration in ready-to-eat meat products, given its complex lipid–protein matrix, seasoning-derived flavor complexity, and susceptibility to oxidative changes during ambient storage—challenges commonly encountered in the commercial meat industry (Sun et al., 2024; Wang et al., 2025; Zhang et al., 2025). The study objectives are to monitor changes in sensory and physicochemical indices during storage, characterize the overall taste and odor profiles using an E-tongue and an E-nose, identify and track the dynamic changes key volatile compounds via GC–IMS, and ultimately establish correlations between chemical composition and sensory quality to identify reliable volatile markers indicative of flavor degradation through a sensory-guided stepwise screening strategy.

## 2 Materials and methods

### 2.1 Materials and sample preparation

High-temperature-sterilized Dezhou braised chicken produced by Dezhou Xiangsheng Food Co., Ltd. (Shandong Province, China) was used. All samples were derived from seven independent production batches of commercially available braised chicken, each batch produced using the same standardized breed (817 broiler chickens, 35 days of age) and the same proprietary seasoning formula under identical processing conditions. Sampling was conducted monthly over a six-month period, resulting in samples representative of seven storage durations (0, 1, 2, 3, 4, 5, and 6 months). All samples were stored at 25 ± 1 °C in the dark. Upon completion of sampling across the storage period, a unified analysis using sensory evaluation, physicochemical characterization, E-tongue, E-nose, and GC–IMS was performed on all collected samples to systematically investigate flavor changes during the entire six-month shelf-life.

### 2.2 Sensory evaluation

Sensory evaluation was performed as described previously with minor modifications (Jiao et al., 2022). The sensory panel comprised ten experienced assessors (five men and five women, aged 20–35 years). The panelists were screened for normal olfactory and taste sensitivity and underwent three training sessions to familiarize themselves with the evaluation protocols and product attributes. Panelist performance was validated prior to formal evaluation through triangle tests and replicated evaluations of a reference sample. The overall sensory quality of braised chicken was evaluated using a 9-point hedonic scale (1–9) for color (extremely dull/discolored to vibrant/typical), appearance (unappealing to intact/surface gloss), texture/tissue state (overly soft/tough to tender/juicy), flavor (extreme off-flavor to rich/balanced), odor (rancid/off-odor to pleasant/characteristic), and overall acceptability (dislike intensely to like extremely). Samples (2 × 2 × 1 cm) were randomly coded with three-digit numbers and presented in individual booths under controlled conditions (25 ± 1 °C, 60–70% RH). Each panelist evaluated all samples in triplicate across three separate sessions, rinsing their palate with warm water between the tastings to minimize the carry-over effects. To detail the sensory odor changes in braised chicken during six-month storage, five key odor attributes—chicken aroma, chicken fat aroma, spice aroma, rancid odor, and off-odor—were selected. The panelists were trained following an established sensory analysis methodology (Xu, Wu, et al., 2023; Yao et al., 2024), as detailed in Table S1. The odor intensity was assessed on a 9-point scale ranging from 0 (not detectable) to 9 (extremely intense). The samples were presented in a randomized order under controlled environmental conditions, with each panelist conducting three independent evaluations per sample.

### 2.3 Analysis of physicochemical properties

Physicochemical properties, including color, shear force, total volatile basic nitrogen (TVB-N), thiobarbituric acid reactive substances (TBARS), pH, and TVC, were measured to systematically evaluate changes in the quality of braised chicken during storage. All measurements were performed in six replicates (*n* = 6).

The color parameters (L*, a*, b*) of the braised chicken samples were measured using a chroma meter (CR-400, Konica Minolta, Osaka, Japan), using a previously described method with modifications (Yao et al., 2024). For each sample, three random measurements were performed on the intact skin of the central breast.

The shear force was determined using the modified Warner–Bratzler method (Xiao et al., 2020). Chicken breast and thigh meat samples were trimmed into standard strips of approximately 2 × 1 × 1 cm, with their long axes aligned parallel to the muscle fiber direction. Measurements were performed using a texture analyzer fitted with a Warner–Bratzler triangular shear blade, operating at a pre-test speed of 5.0 mm s^−1^, a test speed of 2.0 mm s^−1^, a post-test speed of 10.0 mm s^−1^, and a trigger force of 5 g. The shear force was expressed as the maximum force (N) required to cut through the sample.

The TVB-N content was determined according to the Chinese National Standard method (GB/T 5009.228–2016). Minced braised chicken (10 g) was mixed with 75 mL of distilled water and allowed to stand for 30 min, after which magnesium oxide (1 g) was added for immediate distillation. Distillation and titration were performed using a fully automated Kjeldahl nitrogen analyzer. The TVB-N content was expressed as milligrams per 100 g of sample (mg/100 g).

Thiobarbituric acid reactive substances (TBARS) were used to assess lipid oxidation (Shi et al., 2025). Briefly, a homogenized sample (5.0 g) was mixed with 50 mL of trichloroacetic acid (TCA) solution, shaken at 50 °C for 30 min, and filtered through a 0.45 μm PTFE membrane. An aliquot (5 mL) of the filtrate was reacted with 5 mL of thiobarbituric acid (TBA) solution at 90 °C for 30 min. After cooling, the absorbance was measured at 532 and 600 nm. The net absorbance was quantified against a standard curve prepared using 1,1,3,3-tetraethoxypropane (TEP). The results are expressed as milligram malondialdehyde (MDA) equivalents per kilogram of the sample.

The pH was determined using a previously described method with modifications (Lei et al., 2022). An accurately weighed sample (5 g) was homogenized in 45 mL of distilled water at room temperature for 30 s using a high-speed homogenizer. The pH of the homogenates was directly measured using a calibrated precision pH meter (PHS-3C; Shanghai Leici Instrument Co. Ltd., Shanghai, China).

Total viable count (TVC) was determined according to the Chinese National Standard method GB 4789.2–2022. Briefly, 25 g of sample was homogenized with 225 mL of sterile saline in a stomacher bag for 2 min. Ten-fold serial dilutions were prepared, and 1 mL aliquots were spread on plate count agar and incubated at 36 ± 1 °C for 48 h.

### 2.4 Analysis by E-tongue

The taste characteristics of the braised chicken during its shelf**-**life were evaluated using the E-tongue sensor system TS-5000Z (Insent Inc., Fukuoka, Japan), according to a previously described method with slight modifications (Liu et al., 2024). The sensor array consisted of six taste-specific sensors, C00 (bitter), AAE (umami), CA0 (sour), AE1 (astringent), CT0 (salty), and GL1 (sweet), along with two reference electrodes. For sample preparation, 30.0 g of braised chicken was homogenized with 150 mL of deionized water in a 50 °C water bath for 30 min. The homogenate was centrifuged at 3000 ×*g* for 10 min, and the supernatant was filtered through a qualitative filter paper to remove fat and suspended solids. For sensor maintenance, the cleaning solution consisted of two parts: anion cleaning solution (100 mM HCl + 30% ethanol, *v*/v) and cation cleaning solution (10 mM KOH + 100 mM KCl + 30% ethanol, *v*/v). The reference solution (30 mM KCl + 0.3 mM tartaric acid) was used for sensor conditioning and rinsing. During the measurement, the sensor was first cleaned in a cleaning solution for 90 s, followed by two 120-s rinses in a reference solution. The sensor was then conditioned for 30 s using a reference solution. The detection time for each sample was 30 s. All measurements were performed in triplicate.

### 2.5 Analysis by E-nose

To determine the overall aroma profile of braised chicken samples stored for different periods, a PEN3 E-nose system (Airsense Analytics GmbH, Schwerin, Germany) was used according to the method described by Jin et al. (2021), with minor modifications. According to the sample preparation method (Section 2.1), 5.0 g of the sample was weighed into a 50-mL centrifuge tube and sealed with a four-layer plastic wrap. The samples were equilibrated at ambient temperature for 30 min to achieve consistent headspace composition and then analyzed. The following measurement parameters were employed: sensor self-cleaning time, 80 s; sensor zeroing time, 5 s; sample preparation time, 5 s; detection time, 80 s; and carrier gas flow rate, 400 mL/min. The E-nose system was equipped with ten metal oxide sensors. Their response characteristics are listed in Table S2. Each sample was measured in triplicate to ensure reliability. The sensor responses of the E-nose were recorded for further analysis.

### 2.6 Analysis by GC–IMS

Headspace–gas chromatography–ion mobility spectrometry (HS–GC–IMS) was employed to systematically analyze volatile compounds in braised chicken samples stored for six months. The analysis was performed using a FlavourSpec® GC–IMS system (G.A.S., Dortmund, Germany) equipped with a CTC-PAL 3 static headspace autosampler (CTC Analytics AG, Zwingen, Switzerland) and an MXT-WAX capillary column (15 m × 0.53 mm, 1.0 μm, Restek, Bellefonte, PA, USA). According to a previously described method with minor modifications (Sun et al., 2024), the samples (3.0 g each) were weighed into a 20-mL headspace vial and processed at 80 °C for 15 min before injection to ensure sufficient volatile release from the complex food matrix. The headspace conditions included a splitless injection of 500 μL with the needle maintained at 105 °C. GC analysis was performed using a column temperature of 60 °C and an injector temperature of 80 °C, with high-purity nitrogen (≥ 99.999%) as the carrier gas. The flow program consisted of an initial rate of 2.0 mL/min held for 2 min, which was increased linearly to 10.0 mL/min over 8 min and then to 100 mL/min over 10 min. The flow program was finally maintained for 10 min, yielding a total runtime of 30 min. The IMS conditions were as follows: ^3^H ionization source; positive ion mode; 53-mm drift tube at 45 °C; electric field strength of 500 V/cm; and drift gas of high-purity nitrogen at 75.0 mL/min. Qualitative analysis was conducted using *n*-ketone C4–C9 standards to establish a retention index calibration curve. Compound identification was conducted by matching retention indices and drift times against both the GC–IMS library and the NIST database using VOCal software (version 0.4.10). Quantitative analysis was performed using the peak area normalization method to determine the relative contents of volatile compounds, with all data obtained from triplicate measurements and considered valid when RSD ≤ 5%.

### 2.7 Data analysis

The data were analyzed using various statistical and chemometric methods. All experimental data were expressed as mean ± standard deviation. Significant differences among samples from different storage periods were determined by one-way analysis of variance (ANOVA), followed by Tukey‘s honestly significant difference (HSD) post-hoc test using IBM SPSS Statistics software (version 26.0; IBM Corp., Armonk, NY, USA). Statistical significance was set at *P* < 0.05. For multivariate analysis, principal component analysis (PCA) was conducted using the SIMCA software (version 14.1, Umetrics, Umeå, Sweden) to visualize the overall discrimination among samples. Orthogonal partial least squares-discriminant analysis (OPLS-DA) was also performed using SIMCA to identify key variables contributing to differences. Heatmap visualization and Spearman's correlation analysis were performed using the online platform Xiantao Bioinformatics Toolbox (https://www.xiantao.love/products) to illustrate the relationships between the volatile compounds and sensory attributes. Euclidean distance and complete linkage were used for both row and column clustering.

## 3 Results and discussion

### 3.1 Sensory characterization of braised chicken during shelf-life

The sensory evolution of braised chicken over six-month storage is presented in Fig. 1. Although all sensory attributes declined during storage, the magnitude of deterioration varied substantially among the different attributes (Fig. 1A). Odor exhibited the most pronounced degradation, with its score decreasing sharply from an initial 8.35 to 6.43 at six months, approaching the statistical rejection threshold of 6.0 (Giménez et al., 2008). Statistical analysis confirmed that this reduction (1.92 points) was significantly greater than that of all other attributes (*P* < 0.05), establishing odor as the most labile and critical factor governing sensory quality loss (Fig. 1B). In comparison, the decline in taste and texture was moderate, with reductions of 1.16 and 1.02 points, respectively. Although taste deterioration was perceptible, its magnitude was statistically less severe than that of odor (Fig. 1B), suggesting that flavor changes might be dominated by aromatic rather than taste components, a hypothesis investigated further via E-tongue and E-nose analyses. In contrast, the appearance and color demonstrated notable stability, decreasing by only 0.65 and 0.48 points, respectively, indicating their strong preservation throughout storage. The trajectory of overall acceptability, declining from 8.30 to 7.02, reflected this composite sensory deterioration, where the accelerated loss of flavor outweighed the relative stability of other attributes in driving consumer rejection. To further examine the relationship between each sensory attribute and overall acceptability, a Spearman correlation analysis was performed using the mean scores at each storage time point. The results showed that odor exhibited the strongest correlation with overall acceptability (*r* = 0.964, *P* < 0.001), followed by taste (*r* = 0.929, *P* = 0.002), texture (*r* = 0.893, *P* = 0.007), appearance (*r* = 0.893, *P* = 0.007), and color (*r* = 0.750, *P* = 0.052). These findings support the conclusion that odor deterioration is the primary factor driving the decline in overall acceptability. This sensory deterioration pattern aligns with the established pathways of quality loss in stored meat products, primarily driven by progressive lipid oxidation and the depletion of volatile flavor compounds (Al-Dalali et al., 2024). The sensory evolution data demonstrated that the rapid and significant degradation of odor is the predominant factor limiting sensory acceptability.

**Fig. 1:**
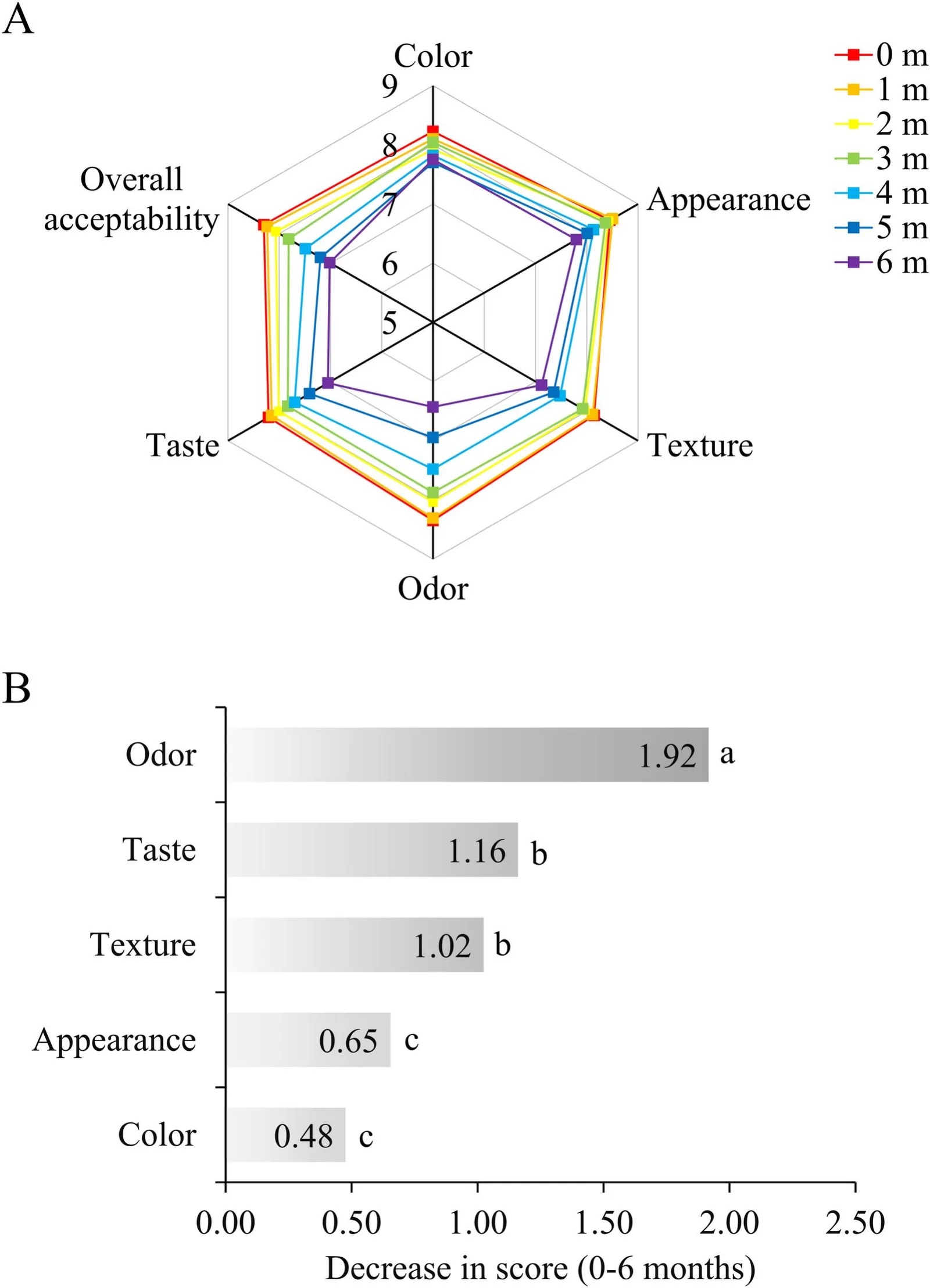
Radar map of sensory evaluation of braised chicken during the six-month shelf-life.

### 3.2 Physicochemical changes in braised chicken during shelf-life

An analysis of the physicochemical results validated the observed decline in sensory scores throughout the shelf-life of braised chicken, particularly the pronounced degradation in odor. The color changes corresponded with the decline in visual appeal (Fig. 2A). The L*, a*, and b* values tended to decrease, with lightness (L*) and redness (a*) declining most noticeably (Fig. 2B). The L* value decreased from 56.25 to 51.62, indicating that the product became visibly darker and lost some of its gloss. The a* value dropped from 10.64 to 8.15, implying that the characteristic reddish-brown color became less intense. The overall color appeared slightly less vibrant. The b* value remained relatively stable, with a slight decrease (from 33.42 to 32.58). Changes in color parameters indicated a slight loss of the bright, reddish appearance, characteristic of the fresh state (Shi et al., 2025; Yao et al., 2024), which corresponded to a modest decline in the sensory scores for color and appearance (Fig. 1). The shear force decreased continuously, indicating excessive tenderness of the meat. The shear force of breast meat dropped from 31.48 to 27.10 N, and that of thigh meat decreased from 29.42 to 26.55 N (Fig. 2C). This textural softening, reflected by the decline in shear force, is consistent with the sensory perception of reduced firmness and chewiness, which directly contributes to the decline in texture scores (Jiao et al., 2022; Xiao et al., 2020).

**Fig. 2:**
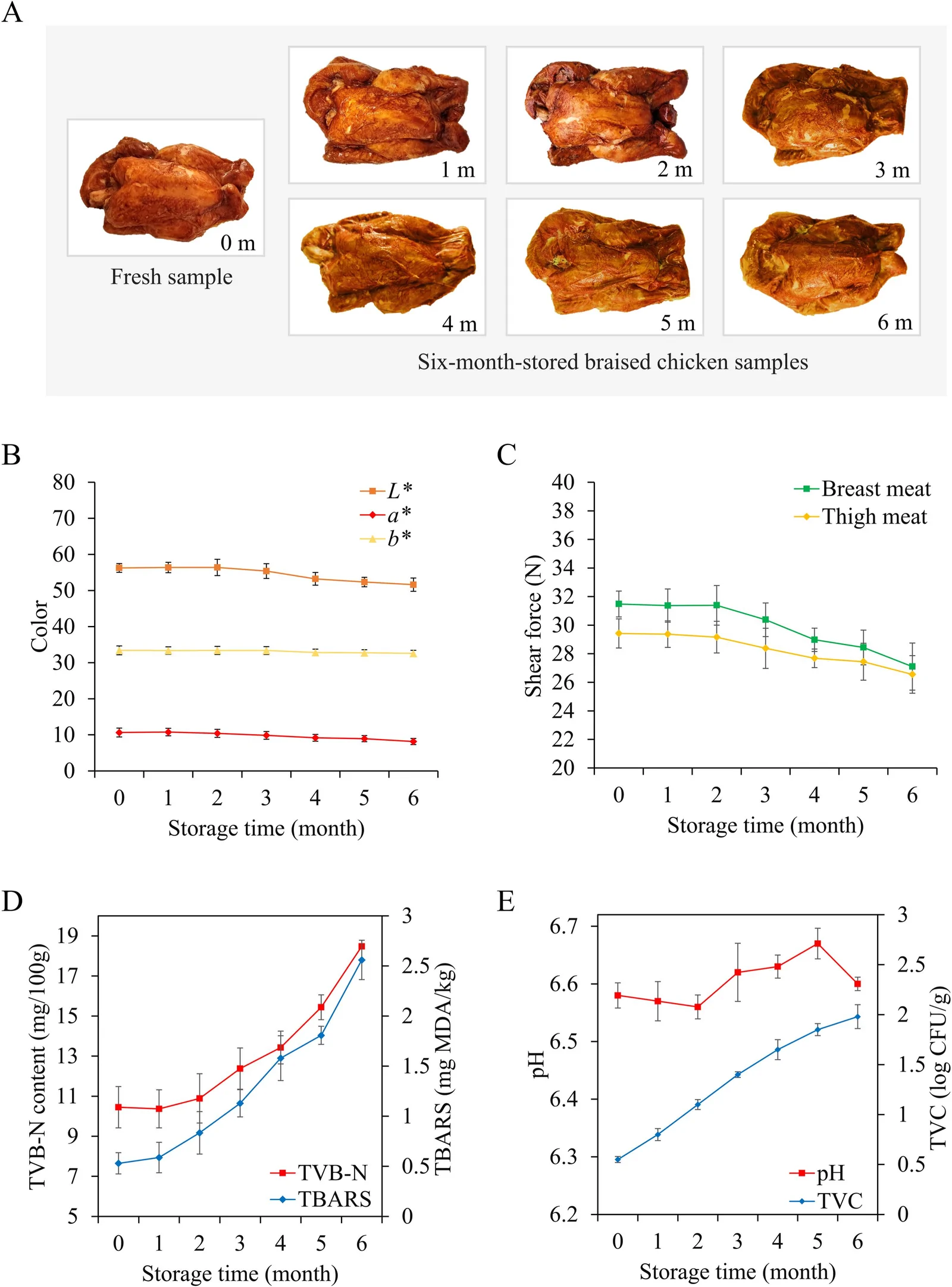
Changes in physicochemical properties of braised chicken during the six-month shelf-life. (A) Appearance. (B) Color (L*, a*, and b*). (C) Shear force. (D) Total volatile basic nitrogen (TVB-N) and thiobarbituric acid reactive substances (TBARS) values. (E) pH value and changes in total viable count (TVC, log CFU/g). Data are presented as mean ± SD (*n* = 6).

Critically, the evolution of key quality deterioration indices directly explains the dramatic odor deterioration. The total volatile basic nitrogen (TVB-N) content increased from 10.45 to 18.48 mg/100 g (Fig. 2D), indicating progressive protein decomposition and the accumulation of alkaline nitrogenous compounds. Notably, these values remained below the sensory rejection threshold of 25–28 mg/100 g for poultry meat (Bekhit et al., 2021), suggesting that protein degradation was progressive but had not yet reached consumer-rejectable levels. Concurrently, thiobarbituric acid reactive substances (TBARS) increased from 0.53 to 2.56 mg MDA/kg (Fig. 2D), signaling advanced lipid oxidation and the generation of aldehydes and ketones that contribute to oxidized and greasy off-flavors (Li et al., 2022; Shi et al., 2025). The concurrent increase in TVB-N and TBARS provides a conclusive physicochemical basis for the severe loss of odor quality that governs the overall sensory rejection. The relative stability of pH (6.56–6.67) during the entire storage period, together with the consistently low total viable counts (below 2.0 log CFU/g throughout storage) (Fig. 2E) suggested that the observed flavor spoilage was primarily driven by the detected chemical reactions rather than by extensive microbial acidification (Lei et al., 2022).

In summary, physicochemical changes confirmed and explained the evolution of the sensory profile. While color darkening and texture softening occurred, the most consequential changes were marked increases in TVB-N and TBARS. These increases directly underpin the accelerated loss in odor quality, affirming it as the predominant factor limiting the acceptability of the product during the six-month storage period.

### 3.3 E-tongue analysis of braised chicken during shelf-life

The taste evolution of braised chicken during storage was objectively quantified using an E-tongue. The radar diagram of the E-tongue (Fig. 3A) indicated a systematic shift in the taste profile during the six months of storage. According to the principle of E-tongue taste interpretation, a sensor response value greater than zero indicates that the taste characteristics contribute to the overall flavor (Hwang et al., 2020). Therefore, the taste characteristics that actually contribute to the overall flavor of braised chicken primarily include umami, richness, saltiness, sweetness, bitterness, and astringency. Umami, richness, and sweetness continuously decreased; saltiness steadily increased; while bitterness and astringency increased during the later stages of storage. As the differences in flavor profiles were not obvious in the radar chart, a bubble chart was employed to highlight the three core indicators that exhibited the most dramatic changes: saltiness, umami, and richness. Data revealed a significant and continuous decrease in the umami (from 11.57 to 11.09) and richness (from 1.42 to 1.04) of braised chicken during storage, contrasted by a steady increase in saltiness (from 2.33 to 3.26) (Fig. 3B). This inverse change caused a noticeable alteration in the overall flavor profile of braised chicken. Studies have indicated that the degradation of Maillard reaction products and lipid oxidation during storage are the main causes of umami reduction in meat products (Xu, Chen, et al., 2023; Xu & Yin, 2024), whereas contrast effects between flavor components can make certain taste characteristics (such as saltiness) more prominent when the overall flavor balance is disrupted (Bolumar et al., 2024). The PCA of the E-tongue data for six-month braised chicken samples showed a clear gradient distribution of samples along the PC1 axis according to the storage time shown in the score plot (Fig. 3C). The cumulative contribution rates of PC1 and PC2 exceeded 80%, indicating that the model reliably presents the primary information of the original data. The loading plot further identified umami, richness, and sweetness as the core contributors to the initial flavor and their continuous decrease as the main driver of flavor evolution. In contrast, saltiness, bitterness, and astringency exhibited a reverse driving effect (Fig. 3D). E-tongue analysis demonstrated flavor degradation in braised chicken during storage, showing a continuous decrease in umami, richness, and sweetness, with a simultaneous increase in saltiness. These changes in key taste indicators corroborate the sensory decline in taste, demonstrating that taste attributes changed during storage in a manner that contributed to the overall flavor deterioration.

**Fig. 3:**
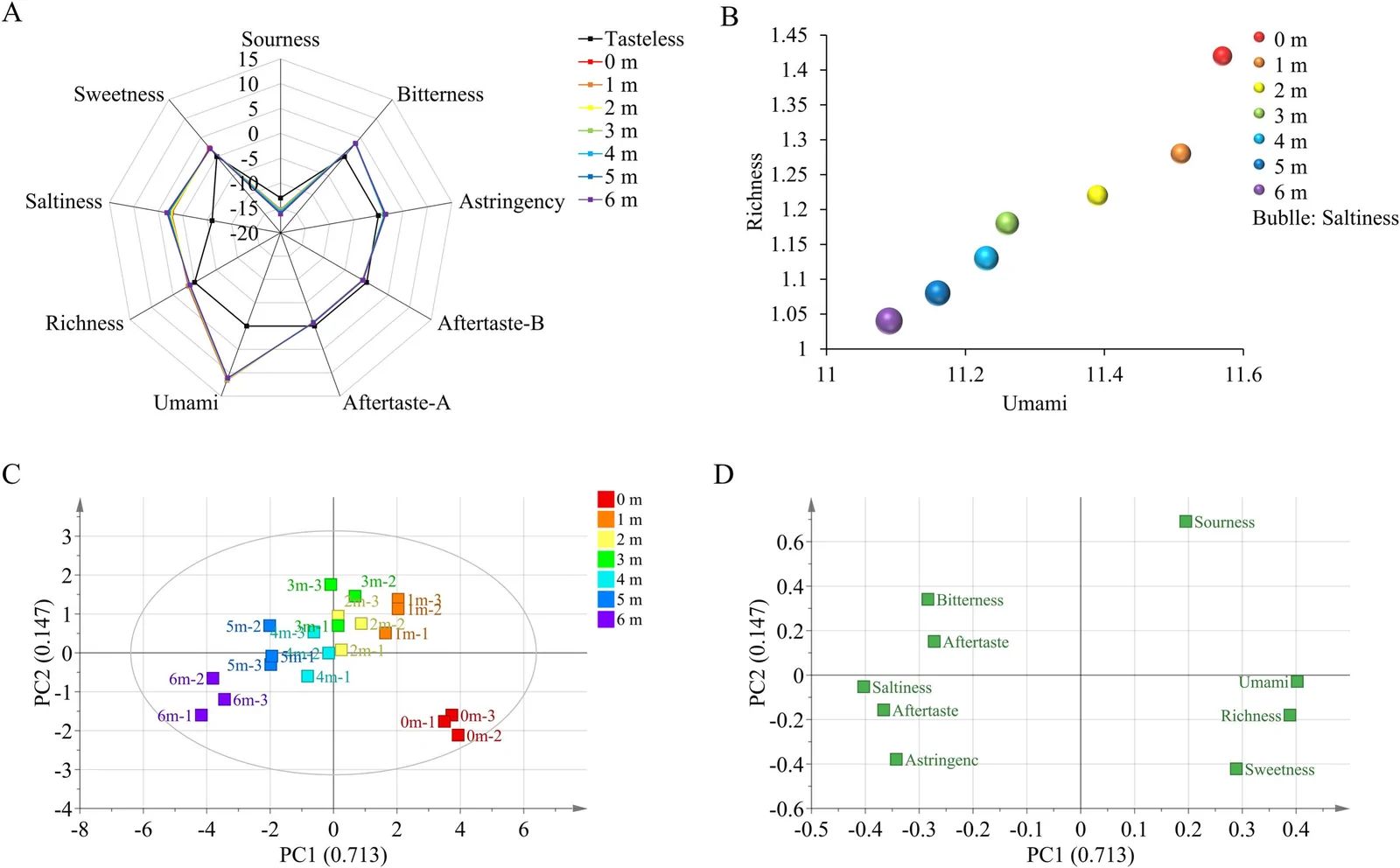
E-tongue analysis of braised chicken during the six-month shelf-life. (A) Radar map of sensor responses. (B) Bubble diagrams of umami, richness, and saltiness. (C) Principal component analysis (PCA) score plots. (D) PCA loading plots.

### 3.4 E-nose analysis of braised chicken during shelf-life

To investigate prominent changes in the overall odor profile implicated by the sensory results, an E-nose analysis was conducted on braised chicken during the entire six-month storage period. According to the radar chart of the E-nose sensor responses (Fig. 4A), its W5S and W1W sensors exhibited high responses to braised chicken products during the six-month shelf-life, followed by W2W, W1S, and W2S. These results suggest the presence of abundant nitrogen oxides, terpenes, inorganic sulfides, aromatic compounds, organic sulfides, short-chain alkanes, alcohols, aldehydes, and ketones in the samples, consistent with the findings of Sun et al. (2024). Over time, the response values of W5S and W1W gradually increased, peaking at six months, suggesting the accumulation of nitrogen oxides and sulfur-containing compounds. These compounds have been commonly observed in meat products during storage (Zhan et al., 2022). Conversely, the decrease in the W1S and W2S responses suggests a reduction in volatiles responsive to these sensors (e.g., short-chain alkanes, alcohols, aldehydes, and ketones), which are typically more abundant in fresh meat products (Yao et al., 2025). The E-nose sensor W2W (aromatic and sulfur compounds) showed an initial decrease, followed by a significant increase during the six-month storage of the braised chicken. This decline in the first four months may reflect gradual changes in aromatic compound profiles, whereas the significant increase in the final two months suggests the accumulation of sulfur-containing volatiles, which are often associated with potential off-odors developed during prolonged storage (Kantono et al., 2021).

**Fig. 4:**
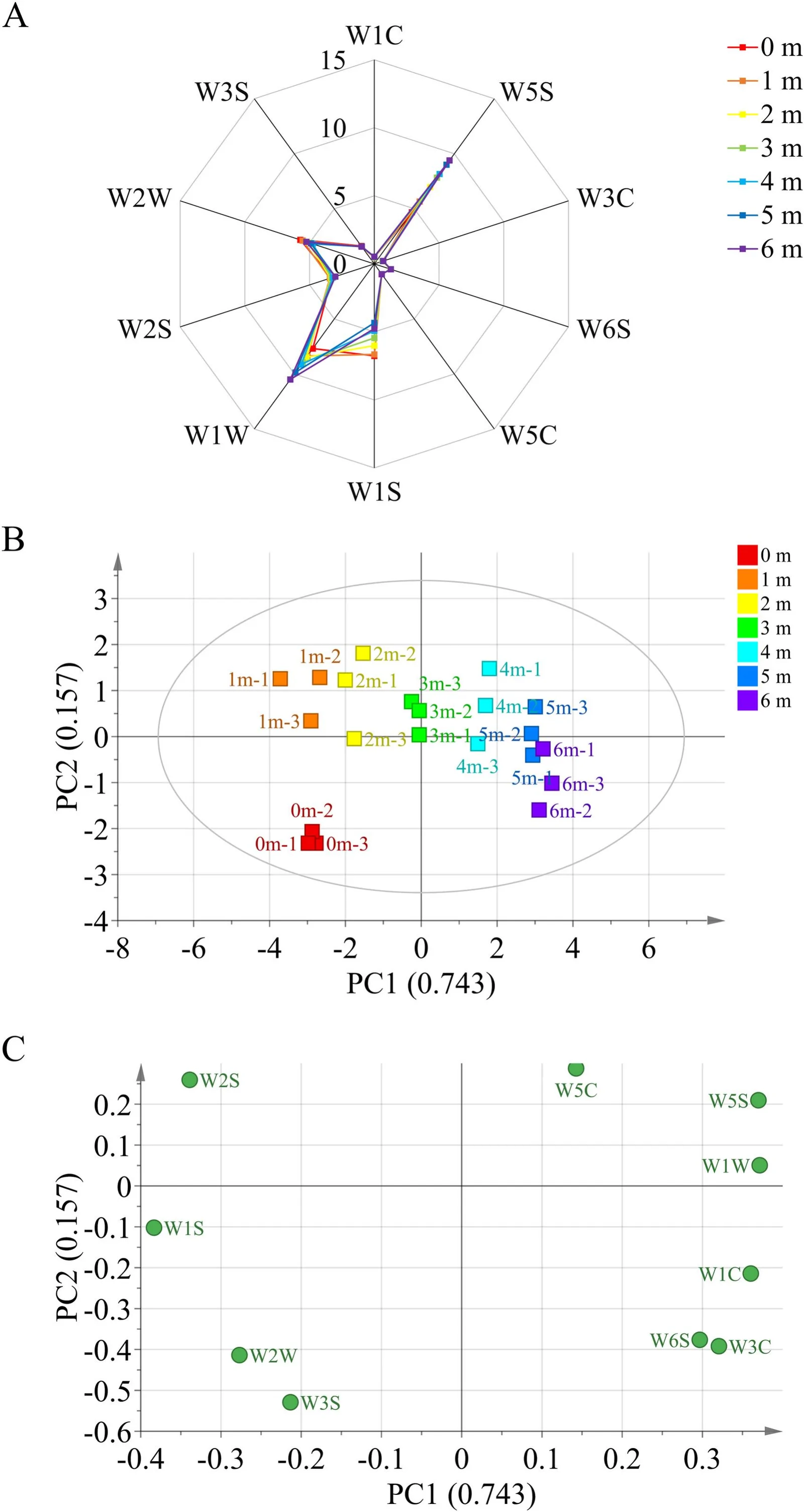
E-nose analysis of braised chicken during the six-month shelf-life. (A) Radar map of sensor responses. (B) Principal component analysis (PCA) score plots. (C) PCA loading plots.

PC1 and PC2 accounted for 74.3% and 15.7% of the variance, respectively, with a cumulative contribution rate of 90.0%, effectively capturing the differences among the samples stored for 0–6 months (Fig. 4B). All later-storage samples (i.e., those stored for 4–6 months) were located in the right quadrant of the PCA score plot, showing a clear separation from the earlier-storage samples along PC1. This result revealed a progressive change in odor characteristics in a time-dependent manner. The PCA loading plot analysis provided crucial insights into the sensor contributions (Fig. 4C). The right quadrant distribution of samples from the later-storage period was positively associated with the W5S and W1W sensors and negatively with the W1S and W2S sensors. Based on the known response characteristics of these sensors (Table S2), this loading plot pattern suggests potential shifts in volatile composition during storage, with the W5S and W1W sensors (sensitive to nitrogen- and sulfur-containing compounds) showing increased associations with later-stage samples, while the W1S and W2S sensors (responsive to alcohols, aldehydes, and ketones) showing decreased associations. These changes in volatile composition align well with the decline in the sensory evaluation of odor during storage, suggesting that an increase in certain volatile compounds and a decrease in others collectively contribute to the deterioration of sensory quality.

### 3.5 GC-IMS analysis of volatile flavor compounds of braised chicken during shelf-life

#### 3.5.1 Characterization of volatile compound profiles by GC–IMS

GC–IMS was employed to elucidate dynamic changes in the volatile compounds present in braised chicken over six months. A total of 75 volatile signals were detected, 67 of which were successfully identified, including monomers and dimers (Table S3). These corresponded to 61 distinct compounds, which were categorized into nine classes: alcohols (11), aldehydes (14), ketones (9), esters (5), acids (3), terpenes (2), organosulfur compounds (5), heterocyclic compounds (4), and others (8). To intuitively compare the overall differences among the volatile compounds, a three-dimensional (3D) topographic plot and a difference comparison topographic plot were generated. In the 3D plot (Fig. 5A), the x-axis represents the drift time (normalized by the reactive ion peak (RIP)), the y-axis represents the GC retention time, and the z-axis represents signal intensity. Each peak corresponds to a volatile compound and its height reflects its relative concentration. Significant changes in the peak intensity were observed with prolonged storage, especially in the later months, indicating substantial alterations in the volatile profile. To more clearly highlight differences between the stored and fresh samples, a difference comparison topographic plot was generated using the 0-month sample as the reference (Fig. 5B). Red areas indicate higher concentrations of compounds in the stored samples than those in the reference, whereas blue areas indicate lower concentrations. The difference map precisely visualizes changes in specific flavor substances, with red and blue areas clearly indicating compound formation and depletion, respectively. To visualize differences in volatile compounds among the samples stored for different durations, a GC–IMS fingerprint was constructed (Fig. 5C). Each row represents all the volatile compounds in one sample, and each column represents the same compound across different samples. The fingerprint clearly demonstrates the evolution of volatile flavor compounds in braised chicken with the increase in storage time. Area “a” primarily reflects the loss of several flavor compounds during the storage of braised chicken. The Maillard reaction and Strecker degradation products during thermal processing (e.g., 2,3-butanedione, 2-methylpyrazine, 3-methyl-2-butenal, 2-methyl-2-hepten-6-one, 1-hydroxy-2-propanone, and 3-hydroxy-2-butanone) underwent continued oxidation or polymerization, leading to the irreversible loss of their characteristic roasted, nutty, and creamy flavor notes (Franklin et al., 2018; Jensen et al., 2011; Lin et al., 2025). Notably, 1,8-cineole—the spice-derived compound—physically dissipated because of its high volatility, which weakens the distinctive herbal and spiced background aroma of braised chicken (Sun et al., 2024). As a valuable sulfur-containing flavor compound formed during thermal processing, 2-furanmethanethiol undergoes oxidative degradation owing to the high reactivity of its sulfhydryl group, resulting in a significant decline in the aroma of roasted meat (Aliani & Farmer, 2005). Area “b” is associated with the formation and accumulation of specific compounds, especially during the final storage month. Aldehydes (*n*-pentanal, (*E*)-2-pentenal, (*E*)-2-heptenal, (*E*)-2-octenal, 1-nonanal, heptaldehyde, and 1-octanal) accumulated notably, primarily owing to advanced lipid oxidation in the mid-to-late storage period (Lee et al., 2025). This phenomenon directly contributes to unpleasant greasy, grassy, waxy, and pronounced oxidative off-flavors (Zhang et al., 2025). Alcohols (1-hexanol, 1-pentanol, 1-octen-3-ol), continuously increase and potentially impart overly strong green, earthy, and mushroom-like musty notes that may mask the original aroma (Lee et al., 2025). Although esters (e.g., methyl pentanoate, acetic acid propyl ester, and butyl pentanoate) can provide fruity notes, their excessive accumulation may lead to aroma imbalance and discordant cloying sweetness and solvent-like off-odors (Uhlig et al., 2024). The lipid oxidation marker 2-pentyl furan is typically associated with enhanced beany and grassy notes when present in large amounts, potentially indicating accelerated deterioration of fat (Stetzer et al., 2008; Xu, L. et al., 2023). Pyrazines showed divergent trends: 2-methylpyrazine decreased by 28.3% (*P* < 0.05), while 2,5-dimethylpyrazine increased by 79.3%. Given its lower odor threshold, the decrease of 2-methylpyrazine likely contributed to the loss of roasted and nutty notes (Xu & Yin, 2024), whereas accumulation of the latter and other nitrogenous compounds supported the increasing E-nose W5S signals during storage. Furthermore, sulfur-containing compounds, such as diethyl disulfide, may transform into undesirable sulfurous and rotten vegetable-like off-odors in later storage stages (Huang et al., 2019; Shi et al., 2025), further exacerbating the overall flavor degradation.

**Fig. 5:**
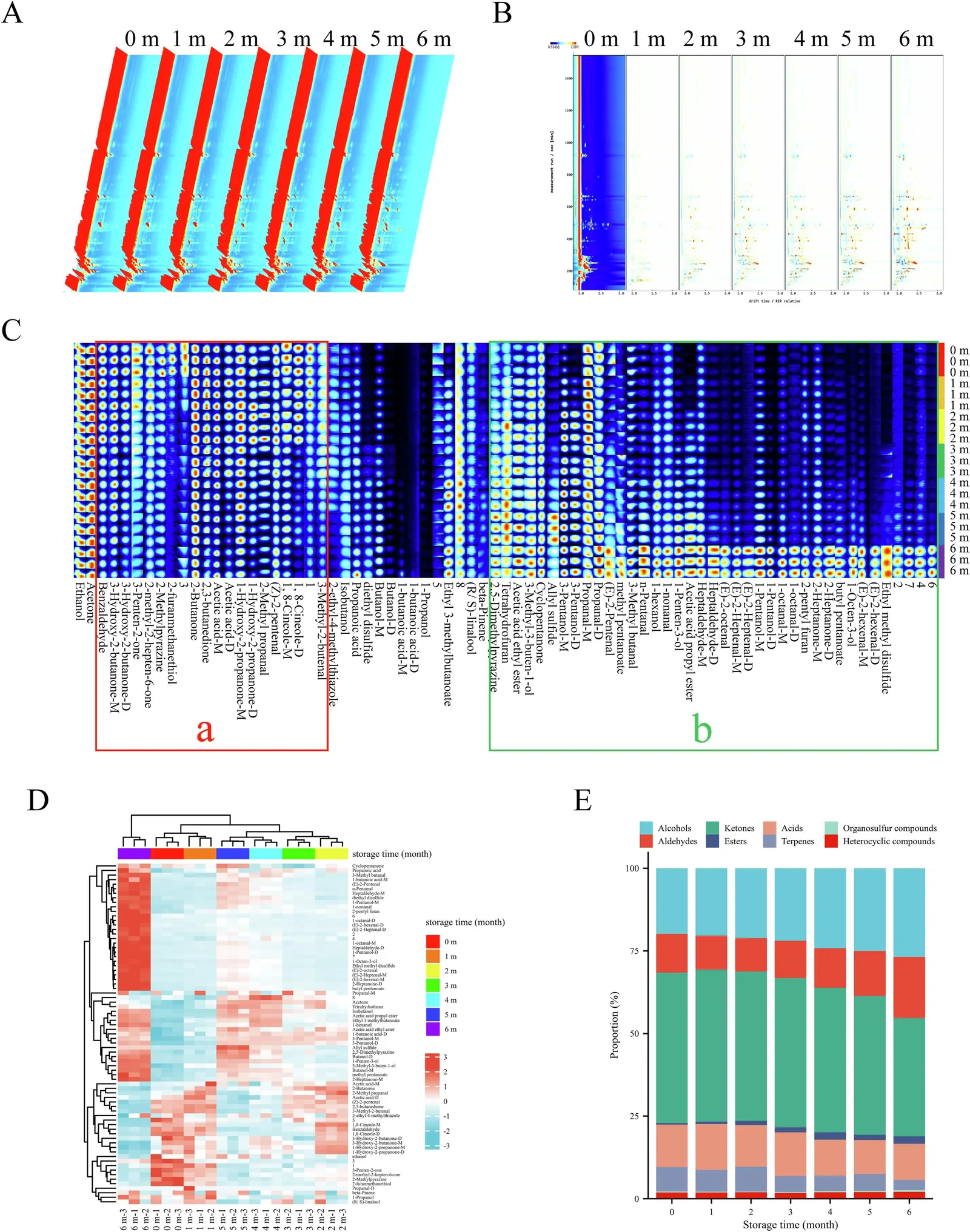
GC–IMS analysis of volatile compounds in braised chicken during the six-month shelf-life. (A) Three-dimensional topographic plot. (B) Difference comparison topographic plot (0-month as reference). (C) GC–IMS fingerprints. Areas “a” and “b” highlight representative regions of volatile compound depletion and accumulation over storage time, respectively. (D) Heat map with hierarchical clustering. (E) A stacked graph of compound categories.

Complementary to the fingerprint plot, which visualizes the presence and relative intensity of individual compounds across samples, the heatmap provides a quantitative and clustered overview, effectively highlighting global concentration trends and categorical differences among the 75 volatile organic compounds during the storage of long-shelf-life braised chicken (Fig. 5D). The color gradient intuitively displays the magnitude of changes in the concentration of these compounds. Hierarchical clustering clearly grouped the samples according to the storage time and compounds with similar change patterns. Samples from later-storage months clustered together and were distinctly separated from fresh and early-stage samples. The results confirmed that alcohols and aldehydes increased most significantly over time. Compounds such as 1-nonanal, 2-pentyl furan, 1-octanal, (*E*)-2-heptenal, (*E*)-2-hexenal, 1-pentanol, and 1-octen-3-ol clustered together, showing a marked upward trend as lipid oxidation products. The clustered increase in these lipid-derived aldehydes and alcohols quantitatively supports the development of oxidative off-flavors. In contrast, key ketones like 3-hydroxy-2-butanone and 1-hydroxy-2-propanone, along with terpenes such as 1,8-cineole, formed another cluster with a consistent downward trend, aligning with the loss of creamy, fruity, and spiced notes. Notably, organosulfur compounds like diethyl disulfide exhibited a sharp increase specifically after six months of storage, highlighting a potential late-stage quality shift. This macro-perspective provided by the heatmap effectively supplements the detailed compound-specific insights obtained from the fingerprint plot, offering a more integrated understanding of the braised chicken flavor profile transition during storage.

To analyze the overall evolution from the perspective of different categories of compounds, a stacked graph was constructed based on the GC–IMS quantitative data (Fig. 5E). The stacked graph analysis indicated that alcohols and aldehydes exhibited the most significant increase over the six-month period, which was directly related to deep lipid oxidation (Lin et al., 2025). In contrast, ketone levels gradually decreased in the later stages, likely because of conversion or volatilization (Yao et al., 2025). Ester- and sulfur-containing compounds showed an overall increase, especially during mid-storage, resulting primarily from esterification reactions, Strecker degradation, and microbial metabolism (Shi et al., 2025). The levels of terpenes consistently declined, leading to a weakened spiced aroma background. This stacked graph analysis further corroborates the dynamic evolution of volatile profiles observed by e-nose (Section 3.4). In summary, the volatile profile transitioned from a processing-derived pattern to a storage-deteriorated pattern, which explains the observed degradation in odor quality. Notably, this transition involves multiple pathways rather than a single mechanism. The late-stage surge of diethyl disulfide and butanoic acid, along with the accumulation of lipid oxidation products, suggests concurrent contributions from protein degradation and oxidative reactions. Meanwhile, the accelerated loss of spice- and Maillard-derived compounds after month 4 indicates that physical volatilization and chemical degradation also play significant roles. These observations point to stage-dependent contributions from different pathways, providing a more comprehensive perspective on flavor deterioration. Mechanistically, these changes can be explained as follows. Lipid oxidation proceeds via free radical chain reactions in polyunsaturated fatty acids, generating primary hydroperoxides that subsequently decompose into secondary volatile aldehydes and ketones, such as octanal and heptanal, which are responsible for rancid and oxidized off-odors (Lee et al., 2025). Protein degradation may involve non-enzymatic chemical degradation pathways, such as thermal decomposition of nitrogenous compounds during sterilization and oxidative deamination of amino acids during storage. These pathways convert muscle proteins into peptides and free amino acids, further yielding volatile nitrogenous compounds that contribute to elevated TVB-N and off-flavor development. The relative contribution of enzymatic versus non-enzymatic pathways to TVB-N accumulation in red meat remains to be clarified (Bekhit et al., 2021). Additionally, the decline in Maillard-derived compounds such as 2-furanmethanethiol and 2,3-butanedione reflects the oxidative degradation of sulfur-containing heterocycles and the inherent instability of certain thermal reaction products during extended storage (Aliani & Farmer, 2005; Xu & Yin, 2024). The consistently low TVC and stable pH throughout storage suggest that microbial contributions to protein degradation were limited in this commercially sterilized and vacuum-packaged product, reinforcing that chemical oxidation is the primary driver of flavor deterioration.

#### 3.5.2 Identification of key differential compounds by OPLS–DA

Supervised OPLS–DA was employed to identify the key volatile compounds most critically linked to storage-induced odor deterioration from the comprehensive volatile profiles obtained by GC–IMS (Zhang et al., 2025). To prioritize compounds with greater sensory relevance, an OPLS-DA model was developed based on the relative odor activity value (rOAV) of the volatile compounds. The rOAV of each compound was calculated based on its relative concentration derived from the GC–IMS peak volumes and its corresponding odor threshold (Table S4), which served as a semi-quantitative indicator of its potential olfactory impact within the overall aroma profile (Zheng et al., 2024). The established OPLS–DA model (Fig. 6A) demonstrated excellent explanatory power and robust predictive ability, with the following key model parameters: R^2^X(cum) = 0.999, R^2^Y(cum) = 0.809, and Q^2^(cum) = 0.551. The score plot revealed a distinct temporal gradient distribution of the samples along the first predictive component (*t*[1]) based on storage time, with good within-group aggregation. This result indicates a strong correlation between the overall aroma profile (weighted by flavor contribution) and storage duration. A permutation test with 200 random iterations was conducted to validate the model and prevent overfitting (Fig. 6B). The results showed that the intercepts of the regression lines for R^2^ and Q^2^ from the permutation test were (0.0, 0.459) and (0.0, −0.796), respectively. The negative Q^2^ intercept (−0.796) confirmed that the original model was not overfitted and was statistically valid (Triba et al., 2015). Based on the OPLS–DA model, the variable importance in projection (VIP) value for each compound was calculated (Fig. 6C). Concurrently, a one-way analysis of variance (ANOVA) followed by Tukey's HSD post-hoc test was used to assess the significance of differences among groups for each compound. Ten key differential volatile compounds were subsequently screened using the following dual criteria: VIP > 1.0 and *P* < 0.05. As listed in Table 1, these key differential compounds were proposed as candidate chemical markers and were mainly associated with the evolution of the volatile profile of braised chicken during storage.

**Fig. 6:**
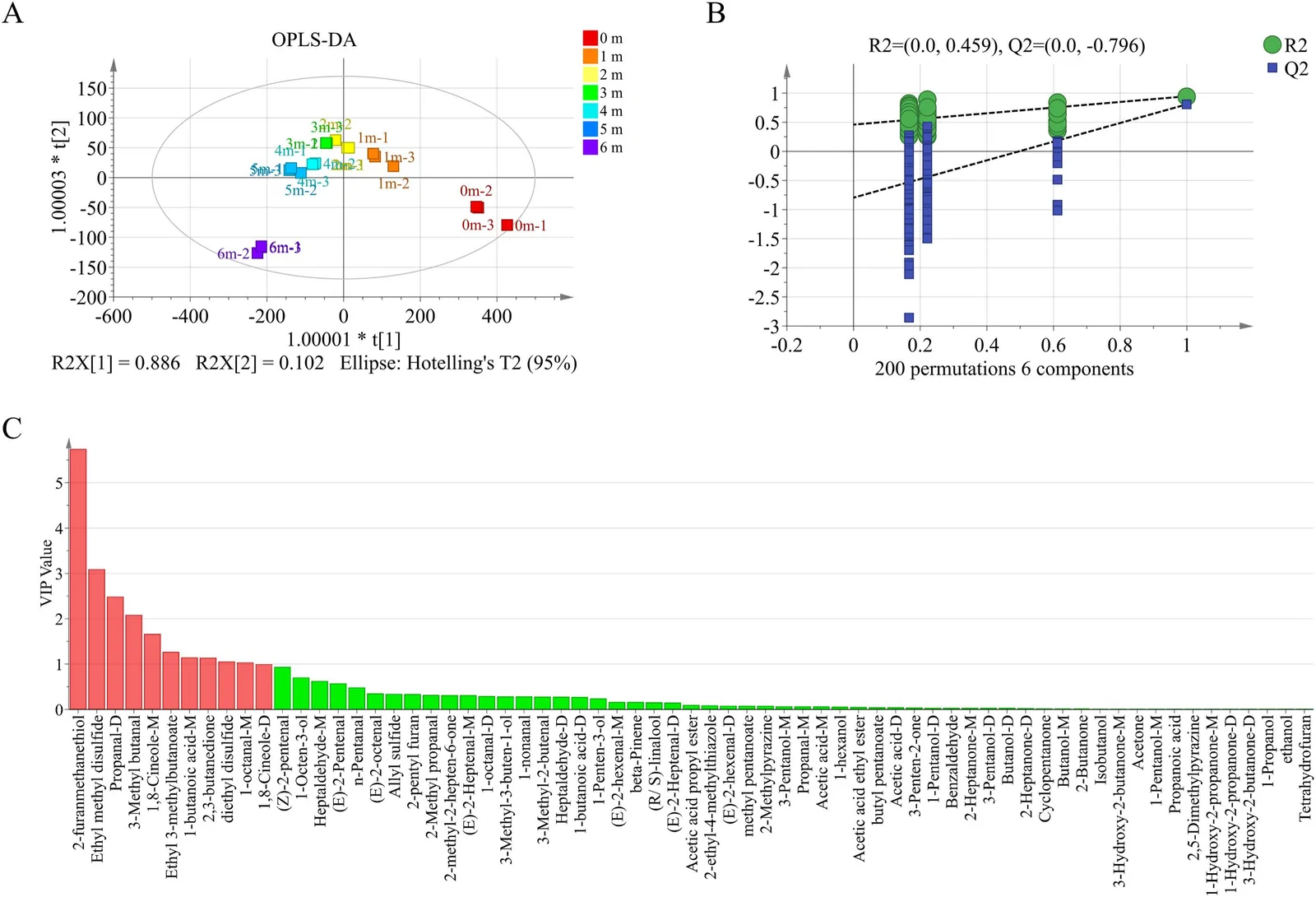
OPLS–DA analysis based on GC–IMS data for braised chicken during the six-month shelf-life. (A) Score plots. (B) Cross-validation plot. (C) Variable importance in projection (VIP) values.

**Table 1:** Key differential volatile flavor compounds in braised chicken during six-month shelf-life identified by GC-IMS.

No.	Key compounds	Odor threshold (μg/kg)	Storage time (month)							Odor description
			0	1	2	3	4	5	6	
1	3-Methyl butanal	0.15	668.34 ± 14.84^cd^	532.57 ± 8.08^e^	638.41 ± 16.01^d^	731.38 ± 27.41^c^	895.86 ± 33.08^b^	833.24 ± 40.84^bc^	1211.44 ± 10.82^a^	Malty, nutty, almond, cocoa (Yu et al., 2023)
2	1-Octanal-M	2	230.95 ± 11.06^e^	272.63 ± 12.47^d^	291.16 ± 6.52^cd^	290.09 ± 12.33^cd^	340.01 ± 2.67^c^	473.77 ± 13.25^b^	941.06 ± 12.34^a^	Fatty, citrus, waxy, green, a slight rancid nuance (Yu et al., 2023)
3	2,3-Butanedione	1.1	2825.16 ± 68.14^b^	3121.81 ± 65.78^a^	2692.13 ± 7.52^bc^	2814.14 ± 37.90^b^	2125.16 ± 4.55^c^	2002.21 ± 10.26^c^	1723.28 ± 4.59^c^	Buttery, creamy, caramel-like (Yu et al., 2023)
4	Ethyl 3-methylbutanoate	6.89	32.17 ± 2.23^d^	100.77 ± 9.98^c^	102.84 ± 4.74^c^	112.29 ± 6.51^bc^	178.52 ± 5.45^a^	142.67 ± 3.36^b^	186.68 ± 6.66^a^	Apple, banana, sour, sweet (Niu et al., 2020)
5	1-Butanoic acid-M	0.23	393.92 ± 14.73^c^	439.79 ± 4.28^c^	473.64 ± 14.05^c^	500.36 ± 23.12^c^	773.33 ± 36.68^bc^	883.44 ± 32.21^b^	1657.07 ± 61.56^a^	Pungent, rancid cheese-like, sweaty, sour (Hansen et al., 2018)
6	1,8-Cineole-M	1.1	4448.61 ± 140.82^b^	4272.28 ± 180.41^b^	5003.79 ± 70.92^a^	3258.22 ± 63.56^c^	3157.77 ± 40.57^c^	3212.97 ± 20.64^c^	2394.11 ± 32.84^d^	Camphor, refreshing herbal, minty, eucalyptus-like (Poitou et al., 2017)
7	1,8-Cineole-D	1.1	586.15 ± 17.06^a^	432.28 ± 13.95^b^	442.23 ± 20.71^b^	197.76 ± 8.35^cd^	185.27 ± 5.44^cd^	217.05 ± 5.80^c^	121.90 ± 5.52^d^	
8	Ethyl methyl disulfide	0.03	3.43 ± 0.56^e^	4.94 ± 1.09^de^	4.36 ± 0.47^de^	5.27 ± 0.78^d^	14.73 ± 1.17^c^	21.38 ± 0.59^b^	65.93 ± 2.16^a^	Pungent, sulfuric, garlic-like, cabbage-like, putrid (Huang et al., 2019)
9	Diethyl disulfide	0.02	31.61 ± 1.52^d^	36.67 ± 1.29^d^	35.35 ± 1.21^d^	60.59 ± 2.65^c^	90.64 ± 4.47^bc^	104.22 ± 5.04^b^	180.52 ± 7.61^a^	Pungent, pong (Huang et al., 2019)
10	2-Furanmethanethiol	0.006	143.25 ± 6.66^a^	92.64 ± 3.51^b^	72.67 ± 3.03^c^	64.16 ± 2.86^cd^	59.45 ± 1.54^cd^	57.14 ± 2.96^cd^	54.44 ± 1.99^d^	Sulfury, coffee, fat, smoky, meaty (Zhu et al., 2021)

Notes: Values are mean ± SD (*n* = 3) of peak volumes (a. u.). M, monomer; D, dimer. Different lowercase letters indicate significant differences among storage months (one-way ANOVA with Tukey HSD post-hoc test, *P* < 0.05).

### 3.6 Correlation between key differential volatiles and sensory odor attributes

To further pinpoint odor markers most closely linked to sensory deterioration, a Spearman correlation analysis was conducted between the ten key differential volatiles (identified in Section 3.5.2) and seven sensory metrics: five refined odor attributes (chicken aroma, chicken fat aroma, spices aroma, rancid odor, and off-odor), the overall odor score, and the overall acceptability score. Temporal changes in these five odor attributes during storage are shown in Fig. 7A. The positive odor attributes, namely chicken aroma, chicken fat aroma, and spice aroma, exhibited a declining trend, decreasing from 8.25 to 7.25, 7.75 to 7.45, and 7.60 to 6.25, respectively. This decline in the desirable aromas during storage aligns with the general pattern of flavor degradation in thermally processed and stored meat products (Lei et al., 2022; Shi et al., 2025). In contrast, the negative odor attributes intensified: rancid odor rose from 0.25 to 1.85 and off-odor increased from 0.60 to 2.25, characteristic of lipid oxidation and microbial spoilage in stored meats (Han et al., 2021; Zhang et al., 2025). This pattern underscores a clear shift from desirable to undesirable odor profiles during storage.

**Fig. 7:**
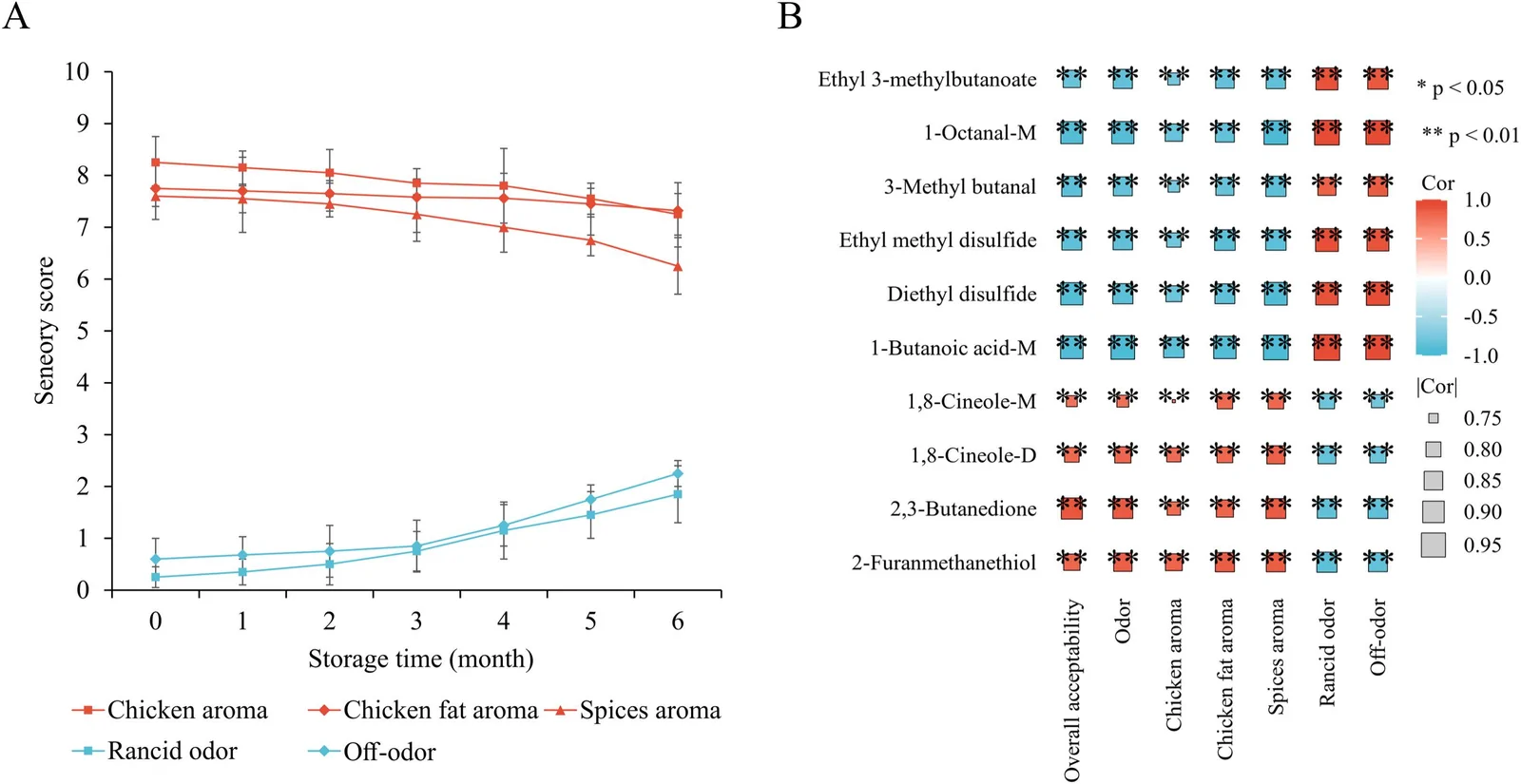
Correlation between odor sensory attributes and key differential volatiles in braised chicken during six-month shelf-life. (A) Changes in odor attribute intensities (chicken aroma, chicken fat aroma, spice aroma, rancid odor, and off-odor) during storage. (B) Spearman correlation heatmap of odor sensory attributes and key differential volatile flavor compounds. Notes: |*r*| > 0.6 and *P* < 0.05 were considered significant; *P < 0.05, **P < 0.01. The color and size of the squares indicate the direction (red: positive, blue: negative) and strength of the correlation, respectively. (For interpretation of the references to color in this figure legend, the reader is referred to the web version of this article.)

The correlation heatmap (Fig. 7B) revealed systematic and significant relationships (*P* < 0.01). First, the three positive odor attributes (chicken aroma, chicken fat aroma, and spice aroma) showed strong positive correlations with the characteristic aroma compounds that declined during storage, such as 1,8-cineole, 2,3-butanedione, and 2-furanmethanethiol. Conversely, they exhibited strong negative correlations with a cluster of off-odor compounds that accumulated during storage, including 1-octanal-M, 3-methylbutanal, diethyl disulfide, and 1-butanoic acid-M. As expected, rancid odor and off-odor—two negative odor attributes—displayed opposite correlation patterns. More importantly, the overall odor score, which integrates the aforementioned five attributes, showed a correlation pattern identical to that exhibited by the positive attributes: strongly negatively correlated with the aforementioned off-odor compounds and positively correlated with the characteristic aroma compounds. Most critically, the overall acceptability exhibited a consistent correlation with odor. This opposing correlation pattern provides mechanistic clarity. This sensory shift can be attributed to two complementary aspects of the same deterioration process. On the one hand, the levels of characteristic pleasant aroma compounds decreased, such as spice-derived 1,8-cineole (Sun et al., 2024) and Maillard-derived 2-furanmethanethiol (Aliani & Farmer, 2005). On the other hand, key off-flavor compounds tended to accumulate, such as octanal, diethyl disulfide, and butanoic acid. These compounds are primarily associated with lipid oxidation, microbial metabolism, and proteolytic degradation (Han et al., 2021; Tatiyaborworntham et al., 2022). This association points to potential intervention targets: the accumulation of lipid oxidation-derived octanal and butanoic acid suggests that antioxidant strategies could help delay flavor deterioration, while diethyl disulfide highlights the need to control microbial activity through appropriate packaging or storage conditions.

Consequently, odor deterioration can be quantitatively defined using specific volatile markers. Among the key differential compounds, four—octanal, diethyl disulfide, butanoic acid, and 2-furanmethanethiol—were identified as pivotal indicators. Similar associations for these compounds have been reported in other stored meat products, supporting their potential relevance as flavor deterioration markers (Han et al., 2021; Shi et al., 2025; Zhang et al., 2025). Their strong and consistent correlations (|*r*| > 0.8) with all seven sensory metrics, most significantly with overall acceptability, indicate that these compounds are closely associated with sensory deterioration in braised chicken during storage. Beyond these four markers, other OPLS–DA-selected compounds generally showed moderate correlations with sensory attributes. Taking 1,8-cineole as an example, its correlation with overall acceptability was 0.762, reflecting its role in the spiced aroma background rather than as a primary driver of overall sensory deterioration. Thus, through this sensory-guided stepwise screening approach, these four compounds have been established as key candidate markers for evaluating the flavor quality of braised chicken during storage. These findings suggest that the four markers not only reflect sensory deterioration but also hold practical value. Octanal and butanoic acid, as indicators of lipid oxidation, point to the potential benefit of antioxidant interventions, whereas diethyl disulfide highlights the importance of controlling microbial activity through antimicrobial packaging or optimized storage. These markers could potentially serve as practical indices for routine quality monitoring in industrial settings.

## 4 Conclusions

This study systematically evaluated the flavor evolution of Dezhou braised chicken during six months of storage. Sensory evaluation identified odor deterioration as the most pronounced effect, with a significantly greater decrease than that in taste, texture, color, and appearance. Physicochemical analyses further corroborated these changes, with significant increases in TVB-N and TBARS (*P* < 0.05), indicating progressive protein degradation and lipid oxidation, respectively, providing a chemical basis for odor deterioration. E-tongue analysis objectively demonstrated a shift in the taste profile toward reduced umami and richness and enhanced saltiness, while E-nose data indicated an overall odor profile enriched with sulfur- and nitrogen-containing compounds. GC–IMS identified 67 volatile compounds, while OPLS–DA screening highlighted ten key differential compounds. Correlation analysis demonstrated strong negative associations (|*r*| > 0.8) between sensory deterioration and four compounds (octanal, diethyl disulfide, butanoic acid, and 2-furanmethanethiol), supporting their role as candidate markers of flavor degradation during the storage of braised chicken. This work establishes a systematic and multidimensional framework for understanding flavor degradation in braised chicken during storage, with direct implications for quality monitoring and shelf-life management of ready-to-eat meat products.

## CRediT authorship contribution statement

**Liu Jing:** Writing – original draft, Visualization, Investigation, Data curation. **Li Huanjia:** Formal analysis, Data curation. **Yue Caishuan:** Writing – original draft, Formal analysis. **Su Zhiwei:** Software, Methodology. **Li Yujin:** Validation, Resources, Project administration. **Wang Bingui:** Writing – review & editing, Supervision. **Guo Yujie:** Writing – review & editing, Funding acquisition.

## Ethical statement

The sensory evaluation in this study followed the relevant ethical guidelines and regulations of China. Ten trained panelists were recruited from Dezhou University and Dezhou Xiangsheng Food Co., Ltd., and provided written informed consent before participation. Participation was voluntary, and panelists could withdraw at any time. No personal identifying information was collected or disclosed. According to institutional and local regulations, this sensory evaluation was exempt from formal ethical review, as it involved only the assessment of commercially available food products and posed no risks to participants.

## Declaration of competing interest

The authors declare that they have no known competing financial interests or personal relationships that could have appeared to influence the work reported in this paper.

## Data Availability

Data will be made available on request.
