# Supplementary material for: Insights into the flavor evolution of braised chicken during six-month storage via sensory evaluation, E-tongue, E-nose, and GC–IMS
Source: Food Chem X. 2026 Oct 15;40:104631. doi: 10.1016/j.fochx.2026.104631 (PMC13691150; doi:10.1016/j.fochx.2026.104631)
Supplement: Supplementary file 1 [file mmc1.docx]

**Caption**

Table S1. List of sensory descriptors for odor.

Table S2. Sensor array composition and typical responses of the PEN3 electronic nose.

Table S3. Volatile organic compounds identified by GC-IMS in braised chicken during the six-month shelf-life.

Table S4. Volatile organic compounds changes in braised chicken during the six-month shelf-life.

**Supplementary Table 1.** List of sensory descriptors for odor.

| Sensory Descriptor | Definition | Scoring Criteria (0-9) | Reference Object | Remarks |
| --- | --- | --- | --- | --- |
| Chicken aroma | Characteristic aroma of cooked chicken | From weak to strong | Cooked chicken breast | Place the skinned chicken breast in a sous-vide bag and cook for 10 minutes. |
| Chicken fat aroma | Aroma of cooked chicken fat | From weak to strong | Cooked chicken fat | — |
| Spices aroma | The complex aroma associated with traditional Chinese spices, characterized by sweet, anise-like, clove-like, and woody notes. | From weak to strong | Spice blend headspace | The spice blend consisted of star anise, Sichuan pepper, cinnamon, fennel, dried tangerine peel, Tsaoko cardamom, nutmeg, and clove, at a ratio of 2:1.5:1:1:1:1:1:1 (w/w). The blend (2 g) was dissolved in 200 mL of hot water for headspace sampling. |
| Rancid odor | Unpleasant aroma from oxidized fats and oils, characterized by fatty, stale, and oxidized off-flavor. | From weak to strong | 100 ppm hexanoic acid in refined sunflower oil | Reference concentrations were set according to AOCS intensity scales to ensure clear panelist recognition and alignment with sample intensities. |
| Oﬀ-odor | The unpleasant aroma associated with microbial spoilage of protein-rich foods, characterized by sweaty, sulfurous, rotten egg, and fecal notes. | From weak to strong | 20 ppm butyric acid in refined sunflower oil | — |

Note: Sensory definitions and reference objects were adapted from Yao *et al.* (2024) and Xu, L. *et al.* (2023). See main text reference list for full citations.

**Supplementary** **Table 2.** Sensor array composition and typical responses of the PEN3 electronic nose.

| Sensor number | Sensor name | Performance description |
| --- | --- | --- |
| 1 | W1C | Sensitive to aromatics ingredients, benzene |
| 2 | W5S | Sensitive to nitrogen oxides |
| 3 | W3C | Sensitive to ammonia and aromatic compounds |
| 4 | W6S | Selective to hydrides |
| 5 | W5C | Sensitive to short-chain alkane, aromatic component |
| 6 | W1S | Sensitive to short-chain alkanes |
| 7 | W1W | Sensitive to many terpenes and inorganic sulfides |
| 8 | W2S | Sensitive to alcohols, ethers, aldehydes and ketones |
| 9 | W2W | Sensitive to aromatic compounds and organic sulfides |
| 10 | W3S | Sensitive to long-chain alkanes |

Note: Sensor array composition and performance descriptions were compiled based on Liu *et al.* (2024) and Sun *et al.* (2024). See main text reference list for full citations.

**Supplementary Table 3.** Volatile organic compounds identified by GC-IMS in braised chicken during six-month shelf-life

| No. | Compounds | CAS | Formula | MW | RI | Rt[s] | Dt[ms] |
| --- | --- | --- | --- | --- | --- | --- | --- |
| Alcohols | |  |  |  |  |  |  |
| 66 | ethanol | C64175 | C2H6O | 46.1 | 940.1 | 215.487 | 1.1401 |
| 69 | 1-Propanol | C71238 | C3H8O | 60.1 | 1040.5 | 286.408 | 1.25371 |
| 51 | Butanol-M | C71363 | C4H10O | 74.1 | 1150.1 | 406.397 | 1.18459 |
| 52 | Butanol-D | C71363 | C4H10O | 74.1 | 1151.3 | 408.123 | 1.38256 |
| 56 | Isobutanol | C78831 | C4H10O | 74.1 | 1103.4 | 346.39 | 1.17637 |
| 38 | 3-Methyl-3-buten-1-ol | C763326 | C5H10O | 86.1 | 1267.7 | 582.228 | 1.16819 |
| 50 | 1-Penten-3-ol | C616251 | C5H10O | 86.1 | 1166.2 | 429.402 | 0.93714 |
| 36 | 1-Pentanol-M | C71410 | C5H12O | 88.1 | 1269.1 | 584.535 | 1.25546 |
| 37 | 1-Pentanol-D | C71410 | C5H12O | 88.1 | 1269.4 | 585.112 | 1.51385 |
| 53 | 3-Pentanol-M | C584021 | C5H12O | 88.1 | 1135.7 | 386.843 | 1.21159 |
| 54 | 3-Pentanol-D | C584021 | C5H12O | 88.1 | 1135.8 | 387.018 | 1.42259 |
| 17 | 1-hexanol | C111273 | C6H14O | 102.2 | 1374.6 | 741.839 | 1.32541 |
| 12 | 1-Octen-3-ol | C3391864 | C8H16O | 128.2 | 1460.9 | 896.992 | 1.15864 |
| 9 | (R/ S)-linalool | C78706 | C10H18O | 154.3 | 1555.7 | 1109.077 | 1.22259 |
| Aldehydes | |  |  |  |  |  |  |
| 71 | Propanal-M | C123386 | C3H6O | 58.1 | 838 | 162.221 | 1.06369 |
| 72 | Propanal-D | C123386 | C3H6O | 58.1 | 839.3 | 162.794 | 1.14136 |
| 67 | 2-Methyl propanal | C78842 | C4H8O | 72.1 | 843.2 | 164.572 | 1.28375 |
| 49 | 3-Methyl-2-butenal | C107868 | C5H8O | 84.1 | 1211.4 | 493.811 | 1.09068 |
| 55 | (Z)-2-pentenal | C1576869 | C5H8O | 84.1 | 1106.5 | 350.018 | 1.09608 |
| 58 | (E)-2-Pentenal | C1576870 | C5H8O | 84.1 | 1141.6 | 394.708 | 1.11193 |
| 68 | 3-Methyl butanal | C590863 | C5H10O | 86.1 | 933.8 | 211.732 | 1.4075 |
| 65 | n-Pentanal | C110623 | C5H10O | 86.1 | 995.9 | 251.853 | 1.42067 |
| 41 | (E)-2-hexenal-M | C6728263 | C6H10O | 98.1 | 1230.7 | 522.547 | 1.17903 |
| 42 | (E)-2-hexenal-D | C6728263 | C6H10O | 98.1 | 1230.6 | 522.4 | 1.51426 |
| 8 | Benzaldehyde | C100527 | C7H6O | 106.1 | 1503.9 | 987.533 | 1.15618 |
| 20 | (E)-2-Heptenal-M | C18829555 | C7H12O | 112.2 | 1341 | 692.309 | 1.25511 |
| 21 | (E)-2-Heptenal-D | C18829555 | C7H12O | 112.2 | 1341 | 692.309 | 1.66769 |
| 45 | Heptaldehyde-D | C111717 | C7H14O | 114.2 | 1192.6 | 467.433 | 1.69308 |
| 46 | Heptaldehyde-M | C111717 | C7H14O | 114.2 | 1191.9 | 466.438 | 1.3379 |
| 14 | (E)-2-octenal | C2548870 | C8H14O | 126.2 | 1428.9 | 834.912 | 1.33096 |
| 32 | 1-octanal-D | C124130 | C8H16O | 128.2 | 1308.1 | 647.121 | 1.81589 |
| 33 | 1-octanal-M | C124130 | C8H16O | 128.2 | 1308.6 | 647.698 | 1.40598 |
| 15 | 1-nonanal | C124196 | C9H18O | 142.2 | 1402.8 | 787.559 | 1.47342 |
| Ketones | |  |  |  |  |  |  |
| 2 | Acetone | C67641 | C3H6O | 58.1 | 890.2 | 166.916 | 1.11771 |
| 26 | 1-Hydroxy-2-propanone-M | C116096 | C3H6O2 | 74.1 | 1323.4 | 667.713 | 1.06017 |
| 27 | 1-Hydroxy-2-propanone-D | C116096 | C3H6O2 | 74.1 | 1323 | 667.149 | 1.22994 |
| 1 | 2-Butanone | C78933 | C4H8O | 72.1 | 919.1 | 205.736 | 1.24081 |
| 73 | 2,3-butanedione | C431038 | C4H6O2 | 86.1 | 1026.2 | 274.89 | 1.18091 |
| 28 | 3-Hydroxy-2-butanone-M | C513860 | C4H8O2 | 88.1 | 1306 | 644.287 | 1.06272 |
| 29 | 3-Hydroxy-2-butanone-D | C513860 | C4H8O2 | 88.1 | 1305.2 | 643.157 | 1.32823 |
| 44 | Cyclopentanone | C120923 | C5H8O | 84.1 | 1193.3 | 468.429 | 1.10477 |
| 63 | 3-Penten-2-one | C625332 | C5H8O | 84.1 | 1135.2 | 386.135 | 1.0679 |
| 47 | 2-Heptanone-M | C110430 | C7H14O | 114.2 | 1186.7 | 459.47 | 1.2628 |
| 48 | 2-Heptanone-D | C110430 | C7H14O | 114.2 | 1188.6 | 461.958 | 1.62893 |
| 18 | 2-methyl-2-hepten-6-one | C110930 | C8H14O | 126.2 | 1356.4 | 714.625 | 1.1737 |
| Esters | |  |  |  |  |  |  |
| 3 | Acetic acid ethyl ester | C141786 | C4H8O2 | 88.1 | 911.9 | 196.993 | 1.33578 |
| 64 | Acetic acid propyl ester | C109604 | C5H10O2 | 102.1 | 988.2 | 246.319 | 1.47863 |
| 70 | methyl pentanoate | C624248 | C6H12O2 | 116.2 | 1094 | 335.372 | 1.21613 |
| 59 | Ethyl 3-methylbutanoate | C108645 | C7H14O2 | 130.2 | 1079 | 320.089 | 1.25383 |
| 34 | butyl pentanoate | C591684 | C9H18O2 | 158.2 | 1320.8 | 664.138 | 1.40768 |
| Acids | |  |  |  |  |  |  |
| 10 | Acetic acid-M | C64197 | C2H4O2 | 60.1 | 1471.7 | 918.904 | 1.0548 |
| 11 | Acetic acid-D | C64197 | C2H4O2 | 60.1 | 1472 | 919.451 | 1.1596 |
| 7 | Propanoic acid | C79094 | C3H6O2 | 74.1 | 1552.1 | 1099.988 | 1.11097 |
| 5 | 1-butanoic acid-M | C107926 | C4H8O2 | 88.1 | 1651.2 | 1373.353 | 1.17238 |
| 6 | 1-butanoic acid-D | C107926 | C4H8O2 | 88.1 | 1650.9 | 1372.347 | 1.38861 |
| Terpenes | |  |  |  |  |  |  |
| 61 | beta-Pinene | C127913 | C10H16 | 136.2 | 1118.1 | 364.226 | 1.21468 |
| 74 | 1,8-Cineole-M | C470826 | C10H18O | 154.3 | 1210.3 | 492.301 | 1.2934 |
| 75 | 1,8-Cineole-D | C470826 | C10H18O | 154.3 | 1209.7 | 491.324 | 1.71628 |
| Organosulfur compounds | |  |  |  |  |  |  |
| 57 | Ethyl methyl disulfide | C20333395 | C3H8S2 | 108.2 | 1157.3 | 416.618 | 1.15923 |
| 40 | diethyl disulfide | C110816 | C4H10S2 | 122.2 | 1237.3 | 532.609 | 1.139 |
| 23 | 2-furanmethanethiol | C98022 | C5H6OS | 114.2 | 1438.9 | 853.962 | 1.10154 |
| 60 | Allyl sulfide | C592881 | C6H10S | 114.2 | 1153.1 | 410.585 | 1.12009 |
| 24 | 2-ethyl-4-methylthiazole | C15679126 | C6H9NS | 127.2 | 1363.4 | 724.966 | 1.1552 |
| Heterocyclic compounds | |  |  |  |  |  |  |
| 4 | Tetrahydrofuran | C109999 | C4H8O | 72.1 | 911.5 | 196.293 | 1.22557 |
| 35 | 2-Methylpyrazine | C109080 | C5H6N2 | 94.1 | 1283.1 | 609.051 | 1.08962 |
| 22 | 2,5-Dimethylpyrazine | C123320 | C6H8N2 | 108.1 | 1337.1 | 686.866 | 1.11635 |
| 39 | 2-pentyl furan | C3777693 | C9H14O | 138.2 | 1241.7 | 539.543 | 1.24691 |
| Others | |  |  |  |  |  |  |
| 13 | Unknown 1 | unidentified | * | 0 | 1453 | 881.26 | 1.09077 |
| 16 | Unknown 2 | unidentified | * | 0 | 1413.5 | 806.609 | 1.26251 |
| 62 | Unknown 3 | unidentified | * | 0 | 1136.6 | 388.04 | 1.12009 |
| 19 | Unknown 4 | unidentified | * | 0 | 1350.1 | 705.372 | 1.41607 |
| 25 | Unknown 5 | unidentified | * | 0 | 1330.1 | 677.069 | 1.15705 |
| 30 | Unknown 6 | unidentified | * | 0 | 1318 | 660.388 | 1.56926 |
| 31 | Unknown 7 | unidentified | * | 0 | 1317.8 | 660.1 | 1.27842 |
| 43 | Unknown 8 | unidentified | * | 0 | 1224.3 | 512.814 | 1.05142 |

Note: M, monomer; D, dimer. Compounds labeled Unknown 1–8 are unidentified volatiles with characteristic mass spectra.

**Supplementary Table 4.** Volatile organic compounds changes in braised chicken during six-month shelf-life

| Compounds | Odor threshold (μg/kg) | Peak volume (a. u.) | | | | | | |
| --- | --- | --- | --- | --- | --- | --- | --- | --- |
|  |  | 0 m | 1 m | 2 m | 3 m | 4 m | 5 m | 6 m |
| ethanol | 500000 | 10542.32±402.72 | 10419.33±78.77 | 10234.42±97.65 | 10219.33±114.31 | 10340.60±153.96 | 10478.17±138.72 | 10087.67±49.15 |
| Acetone | 50000 | 14571.33±199.31 | 15209.44±331 | 15868.32±392.10 | 16039.83±137.48 | 16324.67±61.20 | 16286.27±146.58 | 15473.28±175.03 |
| Benzaldehyde | 1000 | 977.33±31.94 | 937.84±52.77 | 947.08±37.99 | 778.33±14.57 | 722.18±11.53 | 668.26±8.89 | 683.67±20.23 |
| 3-Hydroxy-2-butanone-M | 200000 | 1474.33±126.44 | 1459.67±114.61 | 1534.08±4.36 | 1267.33±52.08 | 1432.83±37 | 1234.33±76.54 | 949.16±19.29 |
| 3-Hydroxy-2-butanone-D | 200000 | 336.37±26.27 | 299.42±16.77 | 313.53±10.02 | 207.67±13.46 | 292.68±18.34 | 243.44±18.79 | 232.28±7.75 |
| 3-Penten-2-one | 50 | 252.33±11.02 | 214.26±3.46 | 184.69±5.03 | 175.67±13.65 | 162.06±4.36 | 154.37±3.51 | 148.54±4.16 |
| 2-methyl-2-hepten-6-one | 5 | 168.71±10.87 | 153.41±2.32 | 144.30±1.83 | 141.39±3.67 | 140.48±1.92 | 129.38±3.88 | 130.21±3.95 |
| 2-Methylpyrazine | 100 | 866.66±51.48 | 751.43±19.92 | 720.23±5.29 | 663.67±10.26 | 646.17±17.38 | 637.39±17.64 | 621.29±14.36 |
| 2-furanmethanethiol | 0.006 | 143.25±6.66 | 92.64±3.51 | 72.67±3.03 | 64.16±2.86 | 59.45±1.54 | 57.14±2.96 | 54.44±1.99 |
| Unknown 3 | - | 223.02±10.58 | 109.33±8.86 | 67.68±5.03 | 82.63±5.03 | 80.27±3.06 | 104.14±7.21 | 134.28±10.31 |
| 2-Butanone | 10000 | 7017.35±113.78 | 7754.64±227.21 | 8037.33±104.39 | 7791±218.6 | 7298.24±74.48 | 7165.65±80.03 | 6581.67±140.98 |
| 2,3-butanedione | 1.1 | 2825.16±68.14 | 3121.81±65.78 | 2692.13±7.52 | 2814.14±37.90 | 2125.16±4.55 | 2002.21±10.26 | 1723.28±4.59 |
| Acetic acid-M | 5000 | 5113.72±45.93 | 5499.73±311.71 | 5383.04±145.44 | 5315.32±69.17 | 5084.13±352.15 | 5240.94±278.98 | 4986.81±126.92 |
| Acetic acid-D | 5000 | 1580.26±60.42 | 1991.51±76.52 | 1699.23±140.38 | 1759.59±66.94 | 1044.12±58.07 | 895.16±68.19 | 1137.62±104.09 |
| 1-Hydroxy-2-propanone-M | 20000 | 2146.66±183.13 | 2297.74±151.24 | 2444.86±15.02 | 2158.82±97.69 | 2160.45±78.6 | 1960.96±102.55 | 1598.78±56.56 |
| 1-Hydroxy-2-propanone-D | 20000 | 1071.34±98.53 | 1098.72±86.95 | 1297.95±21.45 | 834.22±68.16 | 876.74±52.48 | 670.91±52.93 | 448.33±37.58 |
| 2-Methyl propanal | 5 | 66.68±2.08 | 93.43±4.51 | 98.42±5.26 | 72.87±3.79 | 69.60±1.53 | 49.57±1.15 | 51.79±0.58 |
| (Z)-2-pentenal | 1 | 301.29±5.55 | 298.36±4.87 | 300.91±9.65 | 287.22±4.08 | 254.75±7.29 | 222.90±7.78 | 212.42±9.42 |
| 1,8-Cineole-M | 1.1 | 4448.61±140.82 | 4272.28±180.41 | 5003.79±70.92 | 3258.22±63.56 | 3157.77±40.57 | 3212.97±20.64 | 2394.11±32.84 |
| 1,8-Cineole-D | 1.1 | 586.15±17.06 | 432.28±13.95 | 442.23±20.71 | 197.76±8.35 | 185.27±5.44 | 217.05±5.80 | 121.90±5.52 |
| Unknown 1 | - | 206.33±7.55 | 202.67±5.71 | 156.33±8.74 | 142.33±6.01 | 154.67±1.15 | 133.33±0.58 | 146.67±7.74 |
| 3-Methyl-2-butenal | 5 | 139.94±2.91 | 146.85±4.36 | 128.75±4.43 | 123.94±3.83 | 101.91±4.77 | 80.75±1.75 | 75.32±3.46 |
| 2-ethyl-4-methylthiazole | 1 | 24.92±1.51 | 24.68±0.88 | 25.01±1.52 | 25.11±0.62 | 24.41±1.07 | 24.62±1.03 | 24.03±2.11 |
| Isobutanol | 1500 | 51.23±1.55 | 53.09±0.14 | 71.96±0.21 | 80.42±2.02 | 92.43±0.22 | 88.92±3.66 | 94.68±2.22 |
| Propanoic acid | 10000 | 342.26±29.46 | 488.35±24.18 | 524.74±33.84 | 544.91±19.57 | 514.31±21.88 | 517.99±40.57 | 721.38±50.71 |
| diethyl disulfide | 0.02 | 31.61±1.52 | 36.67±1.29 | 35.35±1.21 | 60.59±2.65 | 90.64±4.47 | 104.22±5.04 | 180.52±7.61 |
| Butanol-M | 2730 | 441.22±37.88 | 576.75±9.81 | 676.23±12.17 | 759.28±42.08 | 885.83±8.46 | 915.98±11.97 | 1185.34±12.06 |
| Butanol-D | 2730 | 37.79±1.92 | 52.47±3.45 | 67.64±6.26 | 86.36±6.28 | 123.37±4.35 | 207.91±5.09 | 227.05±8.98 |
| 1-butanoic acid-M | 0.23 | 393.92±14.73 | 439.79±4.28 | 473.64±14.05 | 500.36±23.12 | 773.33±36.68 | 883.44±32.21 | 1657.07±61.56 |
| 1-butanoic acid-D | 0.23 | 302.72±21.18 | 352.43±22.43 | 387.12±6.23 | 390.33±4.73 | 389.99±6.21 | 391.25±7.48 | 392.01±6.17 |
| 1-Propanol | 53950 | 532.71±17.82 | 559.43±15.28 | 509.82±23.41 | 510.35±23.52 | 503.86±3.25 | 518.24±8.73 | 551.07±20.16 |
| Unknown 5 | - | 28.38±1.06 | 21.33±1.53 | 22.67±0.58 | 22.43±1.46 | 17.33±1.08 | 19.67±1.15 | 13.29±0.86 |
| Ethyl 3-methylbutanoate | 0.5 | 32.17±2.23 | 100.77±9.98 | 102.84±4.74 | 112.29±6.51 | 178.52±5.45 | 142.67±3.36 | 186.68±6.66 |
| Unknown 8 | - | 771.67±34.21 | 709.33±19.52 | 810.07±7.94 | 826.33±9.02 | 934.26±14.18 | 840.24±11.36 | 775.43±13.53 |
| (R/ S)-linalool | 20 | 151.42±4.16 | 154.02±1.09 | 149.94±7.71 | 149.49±6.53 | 148.54±4.89 | 142.77±2.23 | 150.46±7.45 |
| beta-Pinene | 6 | 54.96±3.33 | 56.66±4.85 | 54.18±4.75 | 53.23±1.15 | 53.34±3.42 | 55.85±2.56 | 55.72±3.34 |
| 2,5-Dimethylpyrazine | 2000 | 53.13±3.48 | 60.56±4.79 | 68.34±1.79 | 71.58±3.24 | 83.42±6.12 | 98.19±3.19 | 95.27±2.81 |
| Tetrahydrofuran | 100000 | 224.44±1.67 | 285.94±27.76 | 360.64±29.21 | 344.62±20.47 | 404.24±18.09 | 430.72±10.53 | 353.15±26.06 |
| Acetic acid ethyl ester | 800 | 175.13±10.51 | 245.98±15.93 | 305.48±28.62 | 272.66±17.98 | 281.15±21.81 | 335.45±14.93 | 330.84±6.83 |
| 3-Methyl-3-buten-1-ol | 50 | 108.66±7.13 | 153.26±3.15 | 149.95±0.49 | 151.90±6.69 | 148.63±2.03 | 174.24±3.74 | 198.15±1.86 |
| Cyclopentanone | 5000 | 233.22±9.43 | 229.66±7.35 | 232.74±1.91 | 205.71±3.35 | 219.91±4.97 | 251.74±3.54 | 249.72±6.21 |
| Allyl sulfide | 0.005 | 26.49±0.48 | 28.92±0.36 | 33.06±0.48 | 43.43±0.62 | 61.15±1.63 | 100.34±3.72 | 66.34±0.71 |
| 3-Pentanol-M | 800 | 486.98±45.23 | 953.26±57.22 | 1525.09±18.64 | 1519.28±45.13 | 1554.72±4.45 | 1498.08±28.81 | 1531.94±79.08 |
| 3-Pentanol-D | 800 | 50.01±1.18 | 133.16±8.11 | 345.09±20.37 | 392.25±3.79 | 403.25±1.88 | 409.25±1.57 | 455.93±12.69 |
| Propanal-M | 30 | 1224.25±18.92 | 1227.48±27.22 | 1228.17±12.01 | 1237.46±18.51 | 1221.59±17.45 | 1223.99±17.65 | 1220.84±27.95 |
| Propanal-D | 30 | 2119.97±30.25 | 2182.87±57.88 | 2122.82±35.79 | 2103.45±18.01 | 2102.79±19.49 | 2097.56±15.22 | 2118.56±40.25 |
| (E)-2-Pentenal | 3 | 83.02±2.34 | 86.09±3.73 | 90.45±2.35 | 96.15±1.33 | 103.31±0.52 | 137.97±7.51 | 187.63±3.87 |
| methyl pentanoate | 5 | 19.58±1.71 | 29.93±1.27 | 37.59±2.16 | 45.11±4.23 | 48.81±1.01 | 53.21±4.96 | 78.19±3.94 |
| 3-Methyl butanal | 0.15 | 668.34±14.84 | 532.57±8.08 | 638.41±16.01 | 731.38±27.41 | 895.86±33.08 | 833.24±40.84 | 1211.44±10.82 |
| n-Pentanal | 20 | 816.35±41.14 | 798.68±49.11 | 1058.57±45.54 | 1283.84±43.09 | 1792.39±86.18 | 2400.20±50.11 | 4066.23±100.06 |
| 1-hexanol | 1500 | 72.33±5.51 | 109.27±5.29 | 154.02±2.65 | 122.67±1.15 | 204.33±2.52 | 156.67±4.93 | 232.28±7.02 |
| 1-nonanal | 5 | 168.52±4.75 | 187.96±4.82 | 198.21±4.58 | 199.53±4.83 | 200.05±3.95 | 219.34±9.41 | 282.92±9.58 |
| 1-Penten-3-ol | 50 | 180.08±7.55 | 314.67±15.14 | 440.67±11.93 | 684.37±20.42 | 1297.67±30.07 | 1548.67±24.01 | 2156.33±30.09 |
| Acetic acid propyl ester | 500 | 41.39±1.53 | 107.33±8.59 | 460.37±42.62 | 634.63±21.43 | 1024.38±55.07 | 1094.41±29.85 | 1236.68±76.38 |
| Heptaldehyde-M | 10 | 606.34±1.87 | 637.63±9.04 | 693.57±6.24 | 755.34±17.84 | 797.21±20.08 | 992.44±13.39 | 1406.76±42.63 |
| Heptaldehyde-D | 10 | 46.43±1.15 | 54.84±2.42 | 61.32±1.39 | 63.51±1.37 | 81.66±4.65 | 135.37±2.12 | 401.69±18.71 |
| (E)-2-octenal | 3 | 37.63±1.06 | 40.16±2.86 | 45.41±3.22 | 52.05±2.07 | 80.51±4.45 | 91.07±4.91 | 263.27±23.21 |
| (E)-2-Heptenal-M | 5 | 71.67±5.79 | 95.86±6.05 | 109.62±8.12 | 128.59±6.26 | 194.82±1.93 | 241.68±19.12 | 651.46±44.28 |
| (E)-2-Heptenal-D | 5 | 11.25±0.92 | 13.55±0.47 | 14.28±0.21 | 14.72±0.22 | 15.84±0.37 | 17.78±1.16 | 64.27±4.92 |
| 1-Pentanol-M | 10000 | 481.89±25.24 | 719.23±28.37 | 879.38±8.92 | 991.89±18.12 | 1412.67±20.56 | 1692.87±18.18 | 2867.94±35.58 |
| 1-Pentanol-D | 10000 | 73.63±12.33 | 108.68±4.13 | 136.08±5.68 | 160.96±6.05 | 309.49±13.79 | 438.75±11.43 | 1536.75±25.28 |
| 1-octanal-M | 2 | 230.95±11.06 | 272.63±12.47 | 291.16±6.52 | 290.09±12.33 | 340.01±2.67 | 473.77±13.25 | 941.06±12.34 |
| 1-octanal-D | 2 | 25.47±0.46 | 26.52±0.12 | 26.92±0.25 | 26.38±1.23 | 29.31±0.92 | 38.57±2.95 | 120.52±3.14 |
| 2-pentyl furan | 6 | 120.51±10.75 | 224.22±15.49 | 261.18±18.42 | 257.13±12.18 | 265.18±13.25 | 283.52±20.93 | 607.55±35.49 |
| 2-Heptanone-M | 1000 | 391.24±35.35 | 538.61±44.03 | 619.61±13.97 | 683.99±44.93 | 788.32±13.26 | 799.59±11.66 | 1232.71±41.69 |
| 2-Heptanone-D | 1000 | 48.04±2.72 | 86.81±4.13 | 106.14±7.98 | 127.26±0.81 | 162.34±15.12 | 192.37±8.35 | 649.74±12.76 |
| butyl pentanoate | 50 | 21.22±1.56 | 25.53±1.06 | 38.59±1.42 | 40.77±3.92 | 54.18±2.73 | 63.95±4.23 | 155.91±3.45 |
| 1-Octen-3-ol | 1 | 59.57±1.14 | 82.15±1.07 | 93.63±3.49 | 112.73±1.45 | 231.96±7.16 | 289.37±6.45 | 744.34±14.43 |
| (E)-2-hexenal-M | 30 | 72.38±1.74 | 82.66±0.73 | 85.16±2.05 | 109.21±10.15 | 166.85±2.83 | 256.98±7.19 | 663.33±38.92 |
| (E)-2-hexenal-D | 30 | 17.86±1.09 | 17.82±1.36 | 15.76±1.55 | 17.54±0.34 | 17.71±1.54 | 27.24±0.38 | 109.27±3.95 |
| Ethyl methyl disulfide | 0.03 | 3.43±0.56 | 4.94±1.09 | 4.36±0.47 | 5.27±0.78 | 14.73±1.17 | 21.38±0.59 | 65.93±2.16 |
| Unknown 2 | - | 31.63±2.52 | 31.36±1.53 | 35.68±2.51 | 33.57±0.58 | 40.38±3.06 | 49.29±2.46 | 104.20±6.17 |
| Unknown 7 | - | 103.33±6.17 | 120.62±1.53 | 123.36±6.03 | 112.18±4.58 | 194.20±2.65 | 273.57±9.71 | 803.40±13.75 |
| Unknown 4 | - | 28.32±1.53 | 23.09±2.18 | 31.67±1.03 | 34.74±0.58 | 43.39±2.58 | 38.24±2.65 | 86.26±7.09 |
| Unknown 6 | - | 41.18±2.19 | 31.25±2.65 | 38.33±0.79 | 31.33±1.06 | 35.33±2.37 | 36.48±1.02 | 172.28±5.57 |

Notes: Values are presented as mean ± *SD* (*n* = 3). M, monomer; D, dimer. Data are mean peak area in arbitrary units (a. u.). Compounds labeled Unknown 1–8 are unidentified volatiles with characteristic mass spectra. Odor thresholds (μg/kg) are taken from references (Yu *et al.*, 2023; Niu *et al.*, 2020; Hansen *et al.*, 2018; Poitou *et al.*, 2017; Huang *et al.*, 2019; Zhu *et al.*, 2021). See main text reference list for full citations. “-”, threshold not available.
